# Serum lipids and lipoproteins in malaria - a systematic review and meta-analysis

**DOI:** 10.1186/1475-2875-12-442

**Published:** 2013-12-07

**Authors:** Benjamin J Visser, Rosanne W Wieten, Ingeborg M Nagel, Martin P Grobusch

**Affiliations:** 1 Centre of Tropical Medicine and Travel Medicine, Department of Infectious Diseases, Academic Medical Centre, University of Amsterdam, Meibergdreef 9, PO Box 226601100 DD, Amsterdam, The Netherlands; 2 Centre de Recherches Médicales de Lambaréné (CERMEL), Lambaréné, Gabon; 3 Medical Library, Academic Medical Centre, University of Amsterdam, Amsterdam, The Netherlands; 4 Institute of Tropical Medicine, University of Tübingen, Tübingen, Germany

**Keywords:** Malaria, Lipids, Meta-analysis, Cholesterol, Lipoproteins, High-density lipoprotein (HDL), Low-density lipoprotein (LDL), Very low-density lipoprotein (VLDL), Triglycerides, Haemozoin (Hz)

## Abstract

**Background:**

Serum lipid profile changes have been observed during malaria infection. The underlying biological mechanisms remain unclear. The aim of this paper is to provide an overview on those serum lipid profile changes, and to discuss possible underlying biological mechanisms and the role of lipids in malaria pathogenesis.

**Methods:**

A systematic review and meta-analysis to determine lipid profile changes during malaria was conducted, following PRISMA guidelines. Without language restrictions, Medline/PubMed, Embase, Cochrane Central Register of Controlled Trials, Web of Science, LILACS, Biosis Previews and the African Index Medicus were searched for studies published up to 11 July, 2013, that measured serum lipid parameters in malaria patients. Also, major trial registries were searched. Mean differences in lipid profile parameters were combined in fixed and random effects meta-analysis, with a separate analysis for different groups of controls (healthy, other febrile illnesses or very low parasitaemia). These parameters were also compared between severe malaria and uncomplicated malaria. Funnel plots were used to test for publication bias.

**Results:**

Of 2,518 studies reviewed, 42 met the criteria for inclusion in the qualitative analysis, and of these, 15 reported the necessary data for inclusion in the meta-analysis for cholesterol; nine for high-density lipoprotein (HDL), eight for low-density lipoprotein (LDL), and nine for triglycerides, respectively. Total cholesterol, HDL and LDL concentrations were lower in malaria and other febrile diseases compared to healthy controls. The decline was more pronounced and statistically significant during malaria compared to other febrile diseases. These results were consistent across included studies. Triglycerides were raised compared to healthy controls, but not statistically significant when compared to symptomatic controls.

**Conclusions:**

This meta-analysis suggests that the observed lipid profile changes are characteristic for malaria. Although a definite link with the pathogenesis of malaria cannot yet be demonstrated, plausible hypotheses of biological mechanisms involving host lipid alterations and the pathogenesis of malaria exist. An increased research effort to elucidate the precise pathways is warranted, since this could lead to better understanding of malaria pathophysiology and consequently to novel treatment approaches.

## Background

Patients with malaria often exhibit laboratory abnormalities due to an acute phase response, but little is known about serum lipid profile changes in malaria. In 1978, Lambrecht *et al.*[1] reported transient lipid profile changes in six returning travellers with malaria caused by *Plasmodium vivax* and suggested for the first time that changes in high-density lipoprotein (HDL) and very low-density lipoprotein (VLDL) in human serum are related to the lipid metabolism of the parasite. It was hypothesized that the malaria parasite uses cholesterol and phospholipids from its host, resulting in a decrease of serum HDL. Prior to this report, Angus *et al.*[2-4] utilized lipoprotein electrophoresis in rhesus monkeys infected with *Plasmodium knowlesi* to study serum lipids in malaria. Their results were not conclusive because lipoprotein bands could barely be detected in the serum of controls. Subsequently, several clinical studies showed lipid profile changes in the setting of both uncomplicated and complicated malaria [5-10]. Although the magnitude of changes seems to be related to the severity of malaria in several studies [11,12], others found no correlation between the severity of malaria attacks and the extent of lipid profile changes [13,14]. These transient lipid profile changes in the parasitaemic phase have been suggested by some researchers as a potential adjuvant diagnostic tool for malaria [13,15,16].

Changes in serum lipid profile and lipid metabolism are due to a whole range of at least partially disease-specific mechanisms [17]. The extent of serum lipid profile changes during malaria infection and their underlying biological mechanisms remain unclear. Mechanisms may be partly host related (i e, related to an acute phase reaction [18]), parasite-related [19-21], or a combination of these two. If a link between human host serum lipid alterations and the pathogenesis of malaria can be demonstrated, further studies to elucidate the precise pathways can be conducted. Moreover, novel treatment approaches could be explored with lipid metabolism-regulating drugs. Therefore, it is hypothesized that the lipid profile of malaria exhibits characteristic changes. In addition, it is understood that these changes are specific for the malaria pathogen-host interplay.

### Objectives

The present systematic review aims at identifying serum lipid profile changes in malaria with respect to commonly used laboratory parameters: (total) cholesterol (TC), high-density lipoprotein cholesterol (HDL), low-density lipoprotein cholesterol (LDL), very low-density lipoprotein cholesterol (VLDL) and triglycerides (TG). Also, intermediate density lipoproteins (IDL) and apolipoproteins (all classes) are investigated. Furthermore, different possible underlying biological mechanisms and the role of lipids in the pathogenesis of malaria are discussed.

## Methods

**Figure 1:**
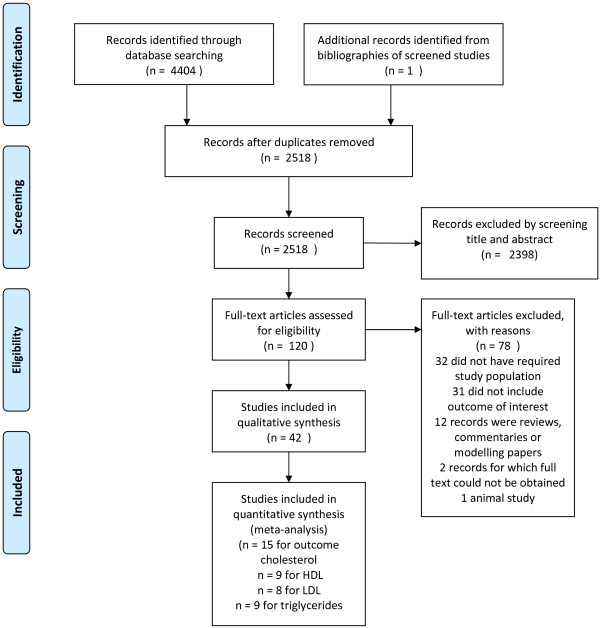
Study selection (PRISMA flow diagram).

Methods of the present review, objectives and inclusion criteria were specified in advance and documented in a protocol (see Additional file 1) [22]. Recommendations made by the Meta-analysis of Observational Studies in Epidemiology (MOOSE) and the Preferred Reporting Items for Systematic Reviews and Meta-Analyses (PRISMA) groups [23-25] were followed. The electronic databases Medline/PubMed (1946 to July 2013), Embase (via Ovid, 1947 to July 2013), Cochrane Central Register of Controlled Trials (The Cochrane Library, 10 July 2013), Web of Science (1975 to July 2013), LILACS (Latin-American and Caribbean Health Sciences Literature; 1982 to July 2013), Biosis Previews (1993 to July 2013) and the African Index Medicus (1993 to July 2013) were searched in order to identify studies published up to present. In addition, major trial registries [26,27] were searched to identify ongoing or future trials. The search strategy consisted of free-text words and subject headings related to malaria and serum lipids. The search strategy was not limited by study design or language. The full search strategies for every searched database are reported in Additional file 2. An experienced clinical librarian (IMN) conducted the actual searches on 9, 10 and 11 July 2013. Bibliographies of relevant studies retrieved from the studies were checked for additional publications. Reference Manager 12.0.3 (Thomson Reuters) was used to manage, de-duplicate and screen the references for eligibility. Reports published before a certain point in time were not excluded. Selection criteria for inclusion of retrieved studies were as follows; the study population consisted of patients with malaria (*Plasmodium falciparum, Plasmodium ovale, P. vivax, Plasmodium malariae* and *P. knowlesi*) of all age groups. Also, at least one of the following outcomes of interest was measured: a) TC; b) HDL; c) LDL; d) IDL; e) VLDL; f) apolipoproteins (all classes); g) TG. All types of studies, including cross-sectional, case–control, case-report and cohort studies were included in the qualitative analysis. To prevent bias, studies with and without (healthy) controls were included. Animal studies were excluded because it is doubtful whether animal studies are comparable to humans regarding lipid profile changes during malaria. Eligibility assessment of studies was performed independently in an unblinded, standardized way by two reviewers (BJV and RWW). Titles and abstracts were screened first, and then one reviewer (BJV) screened and selected relevant full-text articles. For quality control, RWW reviewed 60 (50%) randomly selected full-text articles screened. One author (BJV) extracted the following study characteristics: first author, year of publication, language, study setting, study design, characteristics of trial participants, type and number of controls, type of outcome lipid parameter (TC, HDL, IDL, LDL, VLDL, TG and apolipoproteins) and, if provided, sensitivity/specificity and positive- and negative predictive values. The time of measurement of the outcomes of interest was at admission (“Day 0”) and before anti-malarial treatment. Data was double checked by RWW for all articles included (n = 42). Disagreements in the selection process between reviewers were resolved by consensus or on consultation with the senior author (MPG). The study selection process is summarized in the PRISMA flow diagram (see Figure 1). Authors were not contacted for further information, or to confirm the accuracy of information included in our review with the original researchers, since for the majority of papers adequate contact information was missing. Risk-of-bias assessments for studies included in the quantitative synthesis were made (see Additional file 3). No studies were excluded on the basis of quality.

The existence of publication bias was investigated using funnel plots [28]. A funnel plot is a scatter plot of (treatment) effect against a measure of study size. The risk of publication bias is most probably larger for observational studies than randomized controlled trials, particularly small observational studies as included in this present review [29]. For this reason, the overall publication bias risk for this systematic review is considered as substantial.

### Statistical analysis

Studies that met the eligibility criteria and that reported means and standard deviation (SD), or presented sufficient data for the calculation of means and/or SD, were included in the meta-analysis. The inverse-variance method for the meta-analysis, in which weight is given to each study according to the inverse of the variance of the effect, in order to minimize uncertainty about the pooled effect estimates (see Additional file 4: Statistical analysis). The studies included were allocated into four groups: cholesterol, HDL, LDL, and triglycerides. Studies with healthy controls and symptomatic controls were analysed separately. If studies presented results for uncomplicated as well as for severe malaria [12,30,31], the combined data were used (see Additional file 4). Stratified analyses for uncomplicated and severe malaria were conducted (see Additional file 5). Data were entered by RWW and checked by BJV. Missing data were not problematic since meta-regression of individual data was not done. The I^2^ and 95% CI were used to quantify heterogeneity. I^2^ represents the percentage of the total variation in estimated effects across studies, which is due to real heterogeneity rather than to chance [23]. Initially a fixed effect meta-analysis was performed; however, if I^2^ was large (>50%), which suggests substantial heterogeneity, random-effects analysis was used. Forest and funnel plots were produced to visually assess the mean difference(s) and SD of each study (see Additional file 6). Analyses were done with RevMan 5.2 (Review Manager 5.26. Copenhagen: The Nordic Cochrane Centre, The Cochrane Collaboration, 2012).

## Results

The initial search yielded 4,406 records of which 2,518 remained after removal of duplicates (see Figure 1). Forty-two records [1,5-16,30-57] met the inclusion criteria (Additional file 7: Table S1). Of these, n = 15 for cholesterol [7,9,10,12,30,31,33,35],[38,40,42,44,46,50,52,54]; nine for HDL [10,12,30,33,35,38,42,44],[52]; eight for LDL [10,12,30,33,35,38,42,52]; nine for triglycerides [10,12,30,33,35,40,42,50],[52], contained the necessary data for inclusion in the quantitative analysis (meta-analysis); see PRISMA flow diagram (Figure 1). Four records [11,13,14,47] were excluded from the quantitative synthesis either because means and SD were not reported (n = 3), or mean differences and SD could not be calculated from the available data (n = 1). For one record, a case report [57], the full text was not retrievable. The meta-analysis was done for cholesterol, HDL, LDL and triglycerides (see Additional file 6). For other parameters, such as the apolipoproteins, IDL and VLDL, there was insufficient data to perform a quantitative analysis. If only one study was to be included in the meta-analysis, the forest plot is not shown. For each outcome, two comparisons were made. The first comparison compared means of malaria cases with healthy controls. The second comparison compared malaria cases with symptomatic controls (malaria-like symptoms, but negative for malaria). This second comparison is important as it can indicate whether alterations in lipid profile parameters are malaria specific or also occur in other febrile diseases.

### Description of included studies

For all articles except one [57], the full text paper could be retrieved. The majority of records was published in English; however, 31% (13/42) were in another language (of which French - 19%, 8/42; others 12%, 5/42). Eighty-three percent (35/42) of the records were found using only Medline/PubMed. Fourteen studies took place in sub-Saharan Africa; ten in (Southeast) Asia; 16 in Western countries (Europe, USA, Australia); and two in South America. In total, 3,442 patients with malaria were analysed in these studies, and compared with 1,686 controls (patients negative for malaria). Thirty-five were adult studies and seven were paediatric studies [14,30,31,36,44,52,55]. In 26 of these 42 records, serum lipid profiles of malaria patients were compared to a control group. These control groups consisted of healthy controls (15 studies) [7,9,11,12,14,30,31,33],[38,40,44,47,50] or “symptomatic” controls [16,35,37,44,46] (malaria-like symptoms but negative for malaria). There were two records including controls with a (very) low parasitaemia [10,32]; one of these two [32] did not report the necessary data and was therefore excluded from the meta-analysis; the other study [10] was included in the meta-analysis for healthy controls. A separate analysis was also performed without this study [10]; these results are reported in Additional file 8. In four studies, both healthy controls and symptomatic controls were recruited [13,35,44,46] and compared. In two studies, the controls were not adequately described [41,42]. These two studies were considered as studies with healthy controls. Reference values and measurement units varied considerably among the included studies and were often not reported.

### Cholesterol

**Figure 2:**
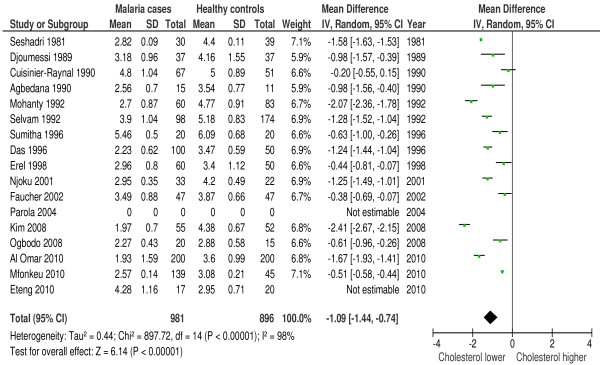
**Forest plot Mean difference for cholesterol (mmol/l) between malaria patients and healthy controls.** Random-effect model.

**Figure 3:**
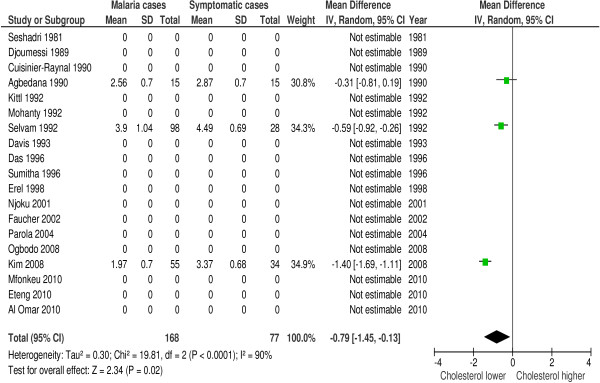
**Forest plot Mean difference for cholesterol (mmol/l) between malaria patients and symptomatic controls.** Random-effect model.

Serum total cholesterol was measured in 36 of 42 included studies. 83% (30/36 studies) reported a hypocholesterolaemia in patients with malaria or a significantly lowered total cholesterol level compared to the control group. Two studies reported a raised cholesterol [36,41], three studies showed no significant differences with the control group [9,39,43] and one study was inconclusive [48] (see Additional file 7: Table S1 for details). Forest plot 1 (n = 15) (Figure 2) shows the mean difference for cholesterol in malaria patients *versus* healthy controls: 1.09 mmol/l or 42.15 mg/dl (95% CI 0.74-1.44 mmol/l), I^2^ = 98%, Z = 6.14 P < 0.00001. Forest plot 2 (n = 3) (Figure 3) shows the mean difference for cholesterol in malaria patients *versus* symptomatic controls: 0.79 mmol/l or 30.55 mg/dl (95% CI 0.13-1.45 mmol/l), I^2^ = 90%, Z = 2.34, P = 0.02. Thus, cholesterol is significantly decreased during malaria. For severe malaria, a separate analysis including three studies [12,30,31] was performed (Additional file 5). Mean difference for cholesterol in severe malaria patients *versus* healthy controls was 1.60 mmol/l or 61.87 mg/dl (95% CI 0.66-2.54), I^2^ = 99%, Z = 3.33, P = 0.0009.

### High-density lipoprotein (HDL)

**Figure 4:**
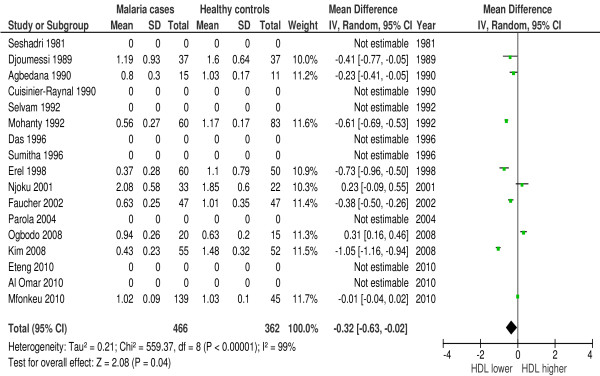
**Forest plot Mean difference for HDL (mmol/l) between malaria patients and healthy controls.** Random-effect model.

**Figure 5:**
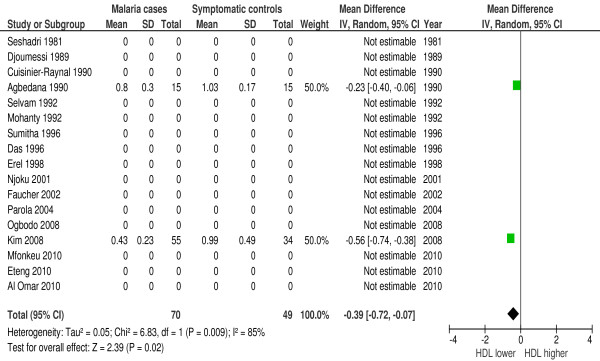
**Forest plot Mean difference for HDL (mmol/l) between malaria patients and symptomatic controls.** Random-effect model.

In 23 of 42 studies HDL was measured during malaria. Eighty-seven percent (20/23 studies) reported a large decline in HDL-concentrations. Forest plot 3 (n = 9) (Figure 4) shows the mean difference for HDL in malaria patients *versus* healthy controls: 0.32 mmol/l or 12.37 mg/dl (95% CI 0.02-0.63 mmol/l), I^2^ = 99%, Z = 2.08, P = 0.04. Forest plot 4 (n = 2) (Figure 5) shows the mean difference for HDL in malaria patients *versus* symptomatic controls: 0.39 mmol/l or 15.08 mg/dl (95% CI 0.07-0.72 mmol/l), I^2^ = 85%, Z = 2.39, P = 0.02. Two studies [30,31] showed a significant larger decline in HDL in severe malaria compared to uncomplicated malaria. Thus, HDL is significantly lower in malaria.

### Low-density lipoprotein (LDL)

**Figure 6:**
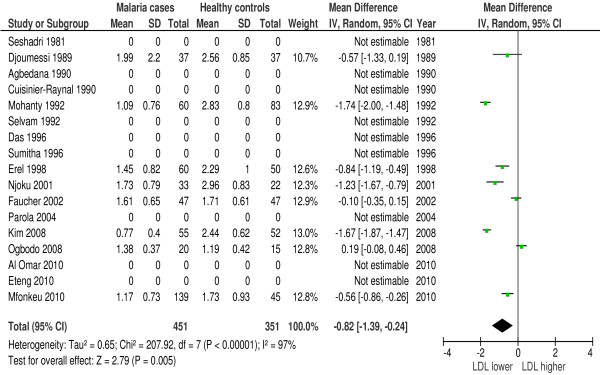
**Forest plot Mean difference for LDL (mmol/l) between malaria patients and healthy controls.** Random-effect model.

In 16 of 42 studies LDL was measured during malaria. Eighty-one percent (13/16 studies) reported a lower LDL-c concentration in malaria patients. Forest plot 5 (n = 8) (Figure 6) shows the mean difference for LDL in malaria patients *versus* healthy controls: 0.82 mmol/l or 31.71 mg/dl (95% CI 0.24-1.39 mmol/l), I^2^ = 97%, Z = 2.79, P = 0.005. Only one study compared LDL in malaria patients with symptomatic controls and found a difference of 1.67 mmol/l or 64.58 mg/dl (95% CI 1.44-1.90 mmol/l), P < 0.01[35]. Two studies [30,31] showed a significant larger decline in LDL in patients with severe malaria compared to patients with uncomplicated malaria. Thus, LDL is significantly lower in malaria.

### Triglycerides

**Figure 7:**
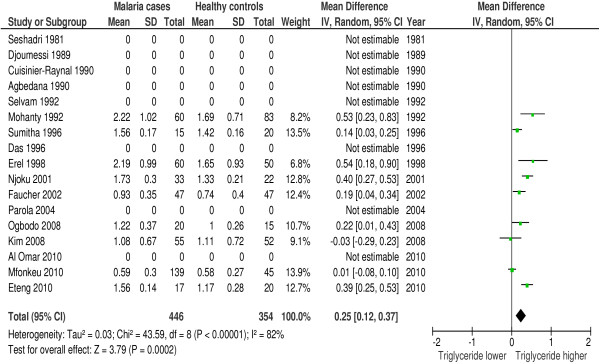
**Forest plot Mean difference for triglycerides (mmol/l) between malaria patients and healthy controls.** Random-effect model.

**Figure 8:**
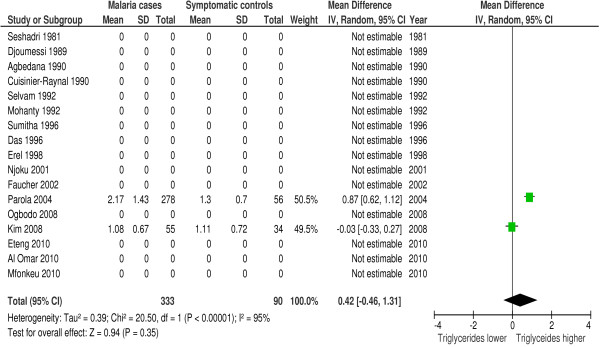
**Forest plot Mean difference for triglycerides (mmol/l) between malaria patients and symptomatic controls.** Random-effect model.

In 23 of 42 studies triglycerides were measured during malaria. Seventy-eight percent (18/23 studies) reported a hypertriglyceridemia and/or a significantly higher mean triglyceride plasma concentration in malaria patients compared to controls. Forest plot 6 (n = 9) (Figure 7) shows the mean difference for triglycerides in malaria patients *versus* healthy controls: 0.25 mmol/l or 22.14 mg/dl (95% CI 0.12-0.37 mmol/l), I^2^ = 82%, Z = 3.79, P = 0.0002. Forest plot 7 (n = 2) (Figure 8) shows the mean difference for triglycerides in malaria patients *versus* symptomatic controls: 0.42 mmol/l or 37.20 mg/dl (95% CI 0.46-1.31 mmol/l), I^2^ = 95%, Z = 0.94, P = 0.35. Thus, triglycerides are significantly higher in malaria patients compared to healthy controls, but these differences become non-significant when compared to symptomatic controls. In patients with severe malaria triglyceride levels were found to be higher compared to triglyceride levels in patients with uncomplicated malaria [12,30].

### Very low-density lipoprotein (VLDL)

Five studies measured VLDL during malaria, of which one case report found marked bands of VLDL [6]. No meta-analysis could be conducted due to insufficient data. In a consecutive case-series in six patients with *P. vivax* malaria, three of six patients had VLDL levels below detection limits [1]. A small cross-sectional study in returning travellers with malaria (n = 12) found a raised plasma VLDL-c in nine of ten patients [8]. This finding was confirmed in another study with 110 malaria patients (both *P. falciparum* and *P. vivax*) with a control group (n = not reported) which found a raised mean concentration compared to the controls (p < 0.05) [41]. An observational study in French soldiers reported “no significant difference found for VLDL” but did not report means and p-values [9].

### Intermediate-density lipoprotein (IDL)

None of the included studies measured or reported intermediate-density lipoprotein (IDL) during malaria.

### Apolipoproteins

Apolipoproteins were measured in five studies. No meta-analysis could be conducted due to insufficient data. A patient with Tangier disease and *P. falciparum* was reported to have apolipoprotein A1 levels below detection levels [34]. A small cross-sectional study with 37 *P. falciparum* patients and 37 healthy controls also found a decrease in apoliprotein A in patients compared to controls, but a higher concentration of apolipoprotein B in malaria patients [38]. Kittl *et al.*[13] observed apolipoprotein A and A-1 lowered in malaria patients, and a very strong correlation with HDL-c (r = 0.96 for Apo A and r = 0.95 for Apo A-1). However, mean values for the malaria group and the control group are not reported. No significant differences between patients and controls for apolipoprotein A1, A2 or B were found by Cuisinier-Raynal [9]; however, mean or p-values were not reported. A pilot descriptive prospective cross-sectional study to investigate the link between nutrition and immunity in Colombian children showed significantly lowered apolipoprotein A1 levels in the malaria group compared to the healthy controls [55].

### Duration of lipid profile changes

The time for malaria patients to recover from the serum lipid profile alterations varied widely across studies. Eight studies [8,10,12,15,34-36,39] reported measurements after “day 0” (admission, before anti-malarial treatment). In most studies, lipid parameters resolved slowly; in one study levels of cholesterol, HDL and LDL were significantly lower in the malaria patients than in the control group at one month after treatment. Most lipid levels had increased at six months while triglyceride levels continued to be lower than normal parameters [35]. These findings contrast with findings from a study in travellers with malaria; both LDL and plasma triglyceride concentrations were normalized at follow up after 2 weeks [15]. In a paediatric study, TC and HDL were still decreased after two weeks, but triglycerides normalized within 14 days. A study with 152 *P. vivax* patients showed that TC, ester and free cholesterol reached normal levels in ten days [46].

### Quality assessment and publication bias

Overall quality assessment scores for risk of bias in studies included in the quantitative analysis ranged from one to five, out of a maximum of five. All studies except one [42] were observational studies. None of them was reported according to all the criteria of the STROBE statement (STrengthening the Reporting of OBservational studies in Epidemiology) [58], a tool in observational studies to prevent bias. In addition, none of the studies that assessed one or more of the lipid profile changes as diagnostic feature of malaria reported the essential information according to the criteria of the STARD statement (STAndards for the Reporting of Diagnostic accuracy studies) [59], a tool to improve the accuracy and completeness of reporting for studies of diagnostic accuracy. Visual assessment of funnel plots showed that the studies were distributed fairly symmetrically about the combined effect size, which suggests little publication bias.

## Discussion

This is the first systematic review and meta-analysis of the impact of malaria on common lipid profile parameters. It confirms previous studies that characteristic serum lipid profile changes occur during malaria. Low serum TC, a low HDL, a low LDL during malaria are described as compared to reference values, healthy and symptomatic controls. Triglycerides were raised during malaria, but this was statistically not significant when compared to symptomatic controls. The conclusion is supported by a similar size and direction of lipid profile changes noted in the records not included in the quantitative synthesis (Additional file 7: Table S1). For IDL cholesterol, VLDL and apolipoproteins, no robust alterations could be observed due to complete absence and paucity, respectively, of studies that measured these laboratory parameters.

The major difficulty regarding the clinically observed serum lipid profile changes is whether they are not only characteristic but truly specific for malaria, rather than a general observed phenomenon that can also be seen in other conditions, particularly (infectious) diseases. Moreover, the association between serum lipid profile changes and malaria is not definitive evidence for the direction of causality, since the existence of confounders, for example, ethnicity, socio-economic status, life-style, food habitats, other infections or diseases, etc., cannot be ruled out and was not corrected for in most of the included studies in this review.

Several arguments support the conclusion that the lipid changes identified are indeed characteristic for malaria and that a causal relationship exists. First of all, several plausible biological mechanisms are at hand (as discussed in some detail below) that can cause these lipid profile changes in malaria patients. Secondly, consistent findings observed by different researchers in different places with different samples are provided in this review (see Additional file 7: Table S1). The fact that data represent studies that included local inhabitants as well as non-immune travellers returning from the tropics, both adults and children, indicates these findings are not ethnic-, age- or geographically specific but observed in a variety of settings and patients (see Additional file 7: Table S1). Results also suggest that a biological gradient is present as greater exposure (severe malaria) leads to greater incidence of the effect (lipid profile changes), as shown in the stratified analysis for severe and uncomplicated malaria (see Additional file 5). An important pre-requisite for causality is temporality: the lipid profile changes have to occur after malaria. This, however, cannot be concluded from this meta-analysis nor the included studies since the majority of the included studies are cross-sectional (cause and effect measured at one specific point of time) or prospective observational studies with malaria present at the start of the study. Inverse temporality is demonstrated; treatment of malaria allows the lipid parameters to normalize. It remains to be elucidated why prolonged lipid profile changes are observed even after parasites have been cleared.

Since only controlled studies were included in the quantitative synthesis, other non-infectious diseases and genetic factors are probably comparable among groups. This supports lipid profile changes as malaria-characteristic features; however, it does not exclude confounding. To investigate the main confounder, namely other infectious diseases, studies that compare lipid profile changes during malaria with control patients that present with other infectious diseases are pivotal. The meta-analysis (Figures 3, 5 and 8) that included comparisons between malaria patients and symptomatic controls suggests that the observed lipid profile changes are indeed specific for malaria. TC, HDL and LDL concentrations were lower in malaria and other febrile diseases compared to healthy controls, however, the decline was more pronounced and statistically significant during malaria. If these lipid profile changes are characteristic for malaria, one could expect more pronounced lipid alterations in severe malaria compared to uncomplicated malaria; this is confirmed by three studies [12,30,42]. Biological mechanisms of lipid profile changes may be partly host-related, i.e., related to an acute phase reaction [18] or parasite-related [20] or a combination of these two.

### Host-related lipid profile changes

Transient plasma lipid profile changes are not only observed during malaria, but also in other acute diseases [60,61]. Typically, HDL and LDL cholesterol levels are slightly reduced, and VLDL levels may be increased. Several researchers demonstrated low cholesterol levels in acute conditions such as surgical trauma [62], malignancy [63], burns [64] and ischemic heart disease [18]. Hence the changes in plasma lipoproteins appear to form part of the acute-phase reaction and can, at least partially, be ascribed to extravasation due to increased capillary permeability [65]. In addition, a decrease of TC and triglycerides has been reported in patients admitted with an acute infection [66,67]. In a study with critically ill patients, the mean HDL level was significantly lower in patients with an infection compared to patients without infection [68]. The TC levels seemed to be slightly lower and triglycerides higher in infected patients, but these differences were not statistically significant [68].

Various forms of lipid disorders have been associated with acute and chronic infectious diseases of different aetiologies: bacterial, viral and parasitological [69-71]. In human immunodeficiency virus (HIV) infection an increase in the levels of triglycerides and reduced levels of cholesterol, HDL-c and LDL-c [72-74] have been observed. Moreover, the treatment of HIV with high-activity, anti-retroviral therapy (HAART) can cause a more atherogenic lipid profile by increased TC, LDL-c and triglycerides [75]. Hypertriglyceridemia has been described in several diseases with haemophagocytosis.

Hypocholesterolaemia has also been described in various haematological diseases [76], including thalassaemia major [77], thalassaemia intermedia [78], sickle cell disease [79], glucose-6-phosphate dehydrogenase (G6PD) deficiency [80], spherocytosis [81], and aplastic anaemia [82]. It can also accompany anaemia with high erythropoietic activity [83]. The pathophysiology of the lowered cholesterol levels in these diseases remains obscure, although several mechanisms have been proposed, including the dilution of serum due to anaemia; increased cholesterol needs associated with erythroid hyperplasia; macrophage activation with the release of cytokines; increased cholesterol uptake by the reticulo-endothelial system; and liver injury secondary to iron overload [83].

### Pathophysiological pathways of lipids during malaria

Lipids are synthesized in the liver, which incidentally happens to be the site where infective malaria sporozoites travel through the bloodstream, invade and take up residence in the hepatocytes. In this asymptomatic ‘exo-erythrocytic stage’ these cells divide until many mature tissue schizonts are formed, each containing thousands of merozoites. The liver schizonts rupture after days and release these merozoites into the bloodstream, initiating the ‘erythrocytic stage’. Within the erythrocyte, a single merozoite divides into eight to 32 merozoites, which demands a considerable amount of lipids (e g, cholesterol) for their anabolic requirements such as membrane formation. The capacity of *Plasmodium* spp*.* to replicate is noteworthy, achieving one of the fastest growth rates among eukaryotic cells [84]. Malaria parasites are intracellular protozoans, auxotrophic and unable to synthesize organic nutrients required for their growth. To ensure their survival and propagation, they must exchange nutrients over the parasitophorous vacuolar membrane (PVM) [85], which surrounds the parasite [86,87]. This PVM neither acidifies nor fuses with organelles of the endocytic cascade and exocytic pathway and is thus actually completely isolated from the host cell vesicular transport system [88].

Keeping this in view, the following questions arise. First, what is the relation between malaria parasites and lipid synthesis in the liver - are malaria parasites capable to produce essential lipids themselves or are host lipids required? Second, does the malaria parasite benefit from high or low serum lipids in the host environment?

In order to meet the nutrients to rapidly multiply within hepatocytes, but more importantly, red blood cells, malaria parasites must scavenge host cell nutrients they cannot synthesize. *Plasmodium* spp. cannot synthesize cholesterol itself [89], similar to other intracellular pathogens. These pathogens most often possess the ability to access this lipid from the exogenous or endogenous pathway of the host [90-93]. Also, no biological pathway involved in sterol production can be demonstrated in *Plasmodium* spp. genome databases [94]. It must be said, however, that morphological data suggest that the parasitophorous vacuole of malaria liver forms does contain sterols [95].

Recent findings demonstrate that the *Plasmodium* genome includes gene-encoding enzymes for phospholipids metabolism [96], allowing *de novo* synthesis of phosphatidylcholine via the Kennedy Pathway (*de novo* synthesis of phosphatidylethanolamine and phosphatidylcholine) and necessitating only the uptake of the small choline molecule [88]. This is important, because these two account for more than 50% of the total phospholipid species in eukaryotic membranes and thus play a major role in the structure and function of those membranes [97]. Moreover, the genome of *P. falciparum* has genes similar to those encoding for the type II fatty acid synthesis pathway in humans. The type II fatty acid synthetic pathway is the principal route for the production of membrane phospholipidacyl chains [98]. These particular genes are embedded within the apicoplast [99], and aid the production of fatty acids, some of which are unique for *Plasmodium* spp*.* [88]. Therefore, *Plasmodium* spp*.* might be able to meet several of its lipid needs from its own biological pathways, even if specific extracellular lipids are necessary for *in vitro* growth [96]. The presence of cholesterol in apicoplast membranes was shown only recently [100].

However, the inability of *Plasmodium* to stock up host molecules makes a continuous supply of nutrients to the parasite necessary [94]. Probably, this is one of the reasons that malaria parasites choose hepatocytes, as they have unique metabolic properties and are especially efficient in internalizing transport proteins (e. g, lipoproteins) via membrane receptors and are proficient at metabolizing different compounds (e. g, glucose, lipids etc.) in relatively huge quantities [101-104].

A recent study shows that *Plasmodium* divert cholesterol from the hepatocyte cell until the release of merozoites. Removal of plasma lipoproteins *in vitro* resulted in a 70% reduction of cholesterol content in hepatic merozoites [94]. It was discovered that *Plasmodium* spp. (*P. yoelii* and *P. berghei)* salvage cholesterol that had been internalized by LDL. However, reduced expression of host LDL receptors did not influence liver stage burden. *Plasmodium* is also capable of seizing cholesterol produced by hepatocytes. Pharmacological blockade of host squalene synthase (an enzyme involved in the first step in sterol synthesis) or the down-regulation of the expression of this enzyme by 80% diminished the cholesterol content of merozoites without effect on parasite development [94]. These data suggest that malaria parasites do need sterols for effective replication, but can also adapt to cholesterol-restrictive conditions by using alternative sources in hepatocytes to maintain infectivity [94]. Another study demonstrated that HDL is essential for the maintenance of *P. falciparum* in *in vitro* culture [105,106]. At relatively low concentrations (0.75 mg/ml protein) HDL is able to aid parasite growth and re-invasion in a serum-free system. In higher concentrations (2.4 mg/ml protein), HDL is toxic to the parasite within infected erythrocytes after invasion, causing abnormal maturation and death of trophozoites [105]. Late ring-stage parasites at a parasitaemia of 2% were cultured with HDL or with phosphate buffer (controls). In the HDL-treated group, unfit parasites developed with reduced size, irregular shape, increasing stain density, and haemozoin (malaria pigment) outside the food vacuole [105]. Both host cholesterol synthesized in the endoplasmic reticulum as well as LDL-derived cholesterol are co-transported to the parasitophorous vacuole. Most probably, compensatory activity of the endogenous and exogenous pathways to provide cholesterol to the parasite exists. Furthermore, the parasite could also cause a hypocholesterolaemia in malaria because it utilizes another pathway, that of receptor-mediated endocytosis [107] where cholesterol is extracted from the blood.

### Host lipids in the formation of haemozoin

Besides the role of lipids in the proliferation and metabolism of the parasite, host lipids have also been implicated in the formation of haemozoin *in vivo*[108,109]. Earlier, it has been shown that linoeic acid (a polyunsaturated fatty acid) may be necessary for the dimerization of ferriprotoporphyrin IX (a toxic compound released after the digestion of haemoglobin), the initial step in the production of haemozoin [110]. Haemozoin is the end product of the plasmodial detoxification of free haem that is produced by haemoglobin degradation [20]. Historically, it was thought that haemozoin was an inert waste product of the malaria parasite. However, recent research resulted in the recognition of the importance of haemozoin in different aspects of malaria [20,111,112]. Haem crystalization is the target of the widely used anti-malarial aminoquinoline drugs [113]. Moreover, not only does the haemozoin production require host lipids, but it appears also that the inhibition of host monocyte functions, one of the eminent immune-modulating haemozoin effects, is caused by hydroxyl fatty acids, generated by *Plasmodium* spp. in large amounts in the human hosts. The lipid hypothesis postulates that haemozoin formation occurs most rapidly at lipid-water interfaces. In the past 3 three years, convincing evidence is emerging in favour of the lipid model. First, the lipid environments in a parasitized erythrocyte using Nile Red (a lipophilic stain), were characterized [114]. Neutral lipids associated with the digestive vacuole of the parasite were observed. These were composed of di- and triacylgycerols (triglycerides); possibly storage organelles for lipid intermediates produced during the degradation of phospholipids in the food vacuole. Mono-, di- and triacylglycerol heterogeneous mixtures promote haemozoin formation, implying that these neutral lipids are involved in haem detoxification [114]. It was demonstrated that triglycerides are a major lipid portion stored in lipid droplets in the late trophozoite and schizont stage of *P. falciparum*. Besides haem detoxification, it may be utilized to store acryl groups for phospholipid synthesis, glycosyl phosphatidyl inositol (a glycolipid) synthesis, and possibly for beta-oxidation (the process by which fatty acid molecules are broken down in the mitochondria to generate acetyl-coA) [115]. Second, another study demonstrated that in the unrelated haemozoin-forming organisms *Schistosoma mansoni*, and in the kissing bug, *Rhodnius prolixus* (triatomine vector of Chagas disease), haemozoin formation occurs inside lipid droplet-like particles or in close association to phospholipid membranes (both hydrophobic environments) [116]. Third, it has been reported that the intracellular mechanism of molecular initiation of haemozoin formation occurs within neutral lipid predominant nanospheres, which envelop haemozoin inside *P. falciparum* digestive vacuoles. It was suggested that haemozoin is formed at the interface between the aqueous medium of the food vacuole and the lipid nanospheres [113]. Another study confirmed these findings, as molecular dynamic simulation showed that a precursor haemozoin dimer forms spontaneously in the absence of the competing hydrogen bonds of water, demonstrating that this substance probably self-assembles near a lipid/water interface *in vivo*. Probably, haemozoin nucleation occurs at the digestive vacuole inner membrane, with crystallizations occurring in the aqueous rather than lipid phase, as indicated by cryogenic soft X-ray tomography [117-120]. Thus, lipids mediate synthetic haemozoin formation very efficiently. Further weight is added to this lipid hypothesis by another recent study that demonstrated that haemozoin-associated neutral lipids alone are capable of mediating haemozoin formation at adequate rates under physiologically realistic conditions of ion concentrations to account for haemozoin formation [121]. The combination of these recent insights makes a compelling case for the theory that lipids drive haemozoin assembly.

### Strengths and limitations

This review triangulates data from quantitative, qualitative and mixed method studies to increase the content validity and comprehensiveness of the review; however, it does not attempt a full analysis of pathophysiological qualitative data [122]. The meta-analysis of lipid parameters was used to explore the effect of malaria between studies and to provide a pooled analysis to support the findings of the narrative (interpretive) synthesis.

A limitation of this meta-analysis is the statistical heterogeneity of the included studies [123,124]. Therefore, random effect models were used. This was possible as the included studies were clinically comparable (see Additional file 7: Table S1) and on visual inspection of the graphs the individual trial effects were in the same direction within the majority overlapping confidence intervals [125]. The statistical heterogeneity in the results is a consequence of clinical or methodological diversity, or both, among the included studies. In particular, the heterogeneity may be due to differences between subgroups of studies. Also, data extraction errors are a common cause of substantial heterogeneity in results with continuous outcomes [23]. However, these were minimized by double-checking in the data extraction process. Clinical variation may have resulted in heterogeneity most obviously, patient characteristics. Also, varying precision of the measurements of lipid parameters in the included studies can have contributed to heterogeneity, although these are unlikely to have fully accounted for heterogeneity in the study design. Significant statistical heterogeneity arising from methodological diversity or differences in outcome measurements suggests that the included studies are not all estimating the same quantity, but does not necessarily suggest that the true malaria effect on lipid parameters varies. In particular, heterogeneity associated solely with methodological diversity would indicate the studies suffer from different degrees of bias, which is the case (see Additional file 3). Nevertheless, as none of the confidence intervals contain zero (except the comparison for the outcome triglycerides with symptomatic controls), there is strong evidence that on average there are malaria-specific effects on common lipid profile parameters.

Another limitation is the poor-to-moderate quality of the studies included in this review, which results from the nature of the study design (some observational studies included healthy controls, some controls with other infectious diseases and some did not include controls at all) and incomplete reporting of essential data (randomization procedures, etc.). However, the consistency of results across studies and settings suggest that the findings of lowered TC, HDL and LDL in patients with malaria are robust.

Reference values of lipid parameters may vary widely between countries and populations, however, they did not influence the results since only mean differences were used in the meta-analysis.

The probability of publication bias was judged as substantial. The funnel plots did not identify publication bias. However, it must be noted that, as a rule of thumb, tests for funnel plot asymmetry should be used with confidence only when there are at least ten studies included in the meta-analysis, because when there are fewer studies the power of the tests is too low to distinguish chance from real asymmetry. This was only the case for funnel plot 1 in the review.

For the literature review, the main limitation was the fact that unpublished studies were not searched for, which may have introduced bias [126]. However, by searching clinical trial registries for ongoing or unpublished studies, this risk of bias was considerably diminished [127]. Whilst no restrictions were placed on the language of publication [128], and no studies were excluded on the basis of language, the focus of the majority of the used search engines (Medline/PubMed being the primary source of studies) to date has been on the European family of languages, and predominantly English. Unfortunately one record [57], published in an Indian medical journal, could not be retrieved despite considerable efforts (contacting the author(s), as well as several European libraries). This, however, does not influence the results since the record would not have been included in the quantitative synthesis anyway.

## Conclusion

Particular serum lipid profile changes are a characteristic feature of malaria. Several hypotheses can explain the underlying biological mechanisms. This review highlights the need for further research into these biological pathways, which can provide new knowledge on the pathogenesis of malaria, and thus open avenues to explore novel anti-malarial interventions. For example, an observational study relating these characteristics of the malaria-specific pathogen-host interplay to the amount of haemozoin produced by human-pathogenic *Plasmodium*.

## Abbreviations

HDL: High-density lipoprotein; LDL: Low-density lipoprotein; IDL: Intermediate-density lipoprotein; VLDL: Very-low density lipoprotein; TC: Total cholesterol; TG/TAG: Triglycerides; Hz: Haemozoin; PVM: Parasitophorous vacuolar membrane; PRISMA: Preferred Reporting Items for Systematic Reviews and Meta-Analyses; MOOSE: Meta-analysis Of Observational Studies in Epidemiology.

## Competing interests

The authors declare that they have no competing interests.

## Authors’ contributions

MPG, BJV and RWW conceived the study. BJV, RWW and MPG developed the study design and the outline of the report. IMN and BJV searched the scientific literature and BJV prepared the first draft of the report. BJV and RWW performed the statistical analyses. RWW, IMN and MPG contributed to the outline of the report. All authors read and approved the final manuscript.

## Supplementary Material

Additional file 1 **Protocol systematic review.** Following the PRISMA guidelines, a protocol for the systematic review was drafted in advance. The documents provides the objectives and proposed search strategies for this systematic review and meta-analysis. Click here for file

Additional file 2 **Search strategies.** In this document, all information sources are described including databases with dates of coverage, detailed search strategies, and the last search date. Click here for file

Additional file 3 **Risk-of-bias assessments.** Document with risk of bias assessments for the quality of non-randomized studies included in this meta-analysis. Click here for file

Additional file 4 **Statistical analysis.** The data provided describes the statistical analysis including the inverse-variance methods for combining results across studies (the meta-analysis), calculation of pooled mean and standard deviation and rounding of the data. Click here for file

Additional file 5 **Stratified meta-analysis for uncomplicated and severe malaria.** Document providing results of the meta-analysis (total cholesterol) stratified for uncomplicated malaria and severe malaria. Click here for file

Additional file 6 **Forest plots & funnel plots.** The data provided describes the full results of the meta-analysis for the different lipid parameters investigated. To increase transparency, both *fixed* and *random* effect analyzes are shown in this document. Click here for file

Additional file 7: Table S1 Studies included in the systematic review and meta-analysis. Click here for file

Additional file 8 **Forest plots without the study of Faucher *et al.*** The data provided describes the results of the meta-analysis for the different lipid parameters investigated without one study with a control group consisting of (very) low parasitaemia malaria patients. Click here for file
