# Supplementary material for: Serum lipids and lipoproteins in malaria - a systematic review and meta-analysis
Source: Malar J. 2013 Dec 7;12:442. doi: 10.1186/1475-2875-12-442 (PMC4029227; doi:10.1186/1475-2875-12-442)
Supplement: Additional file 1 — Protocol systematic review. Following the PRISMA guidelines, a protocol for the systematic review was drafted in advance. The documents provides the objectives and proposed search strategies for this systematic review and meta-analysis. [file 1475-2875-12-442-S1.doc]

**Additional File 1:** Protocol systematic review

**Proposed title**: Serum lipids and lipoproteins in malaria – a systematic review and meta-analysis

**Version:** 1.0

**Date:** 1 July 2013

**Contributors & authors:** Visser BJ, Wieten RW, Nagel IM, Grobusch MP.

**Workplace:** Centre of Tropical Medicine and Travel Medicine Academic Medical Centre, University of Amsterdam.

**Background** Serum lipid profiles alterations in the acute phase of infectious diseases are recognized since long and possible owed to a whole range of at least in part disease-specific mechanisms. With regard to malaria, changes in serum lipid profiles have been described. However, the clinical relevance of these alterations in plasma lipid parameters and lipoproteins metabolism in malaria infection remains unclear. Besides this, very little is known about the underlying biological mechanisms involved in these lipid changes related to malaria. Our aim is to describe the (characteristic) alterations in serum lipids and lipoproteins in malaria and their clinical relevance, with respect to the following commonly used parameters: (total) cholesterol; high-density lipoprotein cholesterol (HDL-c), low-density lipoprotein cholesterol (LDL-c), very low-density lipoprotein cholesterol (VLDL-c), apolipoproteins and triglycerides (TG). Furthermore, we will describe the biological mechanisms underlying these lipid alterations.

**Methods** We will conduct a systematic review according to the PRISMA and MOOSE guidelines to determine the characteristics of lipid profile changes in malaria and their clinical relevance. We will search Ovid Medline, Embase, Cochrane Library (including DARE and Central) CINANL, Web of Science, African Index Medicus, LILACS, Google Scholar and Pubmed (non-Medline citations) for studies published up to 1 July 2013 without language restrictions. If feasible, we will also conduct a meta-analysis with available outcome measurements such as; odds ratios, frequencies or mean differences of lipid parameters.

**Objectives**

1) To determine the serum lipid profile changes of (acute) malaria, with respect to (total-) cholesterol, HDL-c, LDL-c, VLDL-c, apolipoproteins, and triglycerides.

2) To describe the biological mechanisms underlying characteristic serum lipid and lipoprotein changes; are they host-related (i.e. acute phase reaction), parasite-related (selective uptake HDL particles, Maurois 1978/Grellier 1991, etc? ; and the haemozoin theory.

**Synopsis systematic review article**

-Abstract

-Introduction malaria

-Introduction lipids and lipoproteins

-Methods section

-Results:

- (characteristic) lipid profile changes in malaria (Table 1; Characteristics of lipid profiles in malaria, overview of included studies in systematic review)

-Duration of lipid profile changes

-Meta-analysis, if possible with the available data: Forest plots; 2 comparisons, if feasible a) malaria vs. healthy patients b) malaria vs. patients with other infectious diseases

-lipid profile changes in the African subcontinent (population studies etc)

-biological mechanisms (host-related, parasite related)

-recommendation for further (clinical) research

-conclusion

*Suggested tables/figures/data:*

- Overview studies with description of lipid profile changes
- Forest plots
- Funnel plots
- Flow diagram PRISMA selection of studies
- Appendix search strategy; inclusion and exclusion criteria
- Appendix other additional information

**Time schedule:**

June 2013: Preparing protocol, reading literature & prepare search strategy

July 2013: Searching the literature. Screening of records by two independent reviewers (Benjamin J. Visser & Rosanne W. Wieten)

August 2013: Data extraction in standardized Word 2003 document form, data-entry, meta-analysis with Review Manager 5 and drafting the manuscript.

September 2013: Finalizing manuscript and submit to peer-reviewed medical journal with open-access option.
