# Supplementary material for: Serum lipids and lipoproteins in malaria - a systematic review and meta-analysis
Source: Malar J. 2013 Dec 7;12:442. doi: 10.1186/1475-2875-12-442 (PMC4029227; doi:10.1186/1475-2875-12-442)
Supplement: Additional file 2 — Search strategies. In this document, all information sources are described including databases with dates of coverage, detailed search strategies, and the last search date. [file 1475-2875-12-442-S2.doc]

**Additional File 2:** Search strategies

Database: **Medline/PubMed 1946 to July 2013**

Date of search: 9 July 2013

| *#* | *Searches* | *Results* |
| --- | --- | --- |
| #1 | ("Malaria"[Mesh] OR malaria*[tiab] OR "Plasmodium"[Mesh] OR plasmodi*[tiab]) AND ("Lipids"[Mesh] OR "Lipid Metabolism"[Mesh] OR "Lipid Metabolism Disorders"[Mesh] OR lipid[tiab] OR lipids[tiab] OR lipoprotein*[tiab] OR apolipoprotein*[tiab] OR chylomicron*[tiab] OR HDL*[tiab] OR IDL[tiab] OR IDLs[tiab] OR LDL*[tiab] OR VLDL*[tiab] OR alphalipoprotein*[tiab] OR betalipoprotein*[tiab] OR phospholipid*[tiab] OR fatty acid*[tiab] OR sterol*[tiab] OR cholesterol*[tiab] OR fats[tiab] OR glyceride*[tiab] OR triglyceride*[tiab] OR lipogenes*[tiab] OR lipolys*[tiab] OR lipoylation*[tiab] OR dyslipidemia*[tiab] OR dyslipoproteinemia*[tiab] OR hyperlipidemia*[tiab] OR lipidemia*[tiab] OR lipemia*[tiab] OR hyperlipoproteinemia*[tiab] OR hypertriglyceridemia*[tiab] OR hypercholesterolemia*[tiab] OR hypercholesteremia*[tiab] OR hypolipoproteinemia*[tiab] OR hypocholesterolemia*[tiab]) | 2149 |
| #2 | #1 NOT (Animals[Mesh] NOT Humans[Mesh]) | 1232 |

Database: **Ovid Embase 1947 to July 2013**

Date of search: 10 July 2013

| *#* | *Searches* | *Results* |
| --- | --- | --- |
| 1 | exp *malaria/ | 52279 |
| 2 | malaria*.ti,ab. | 72831 |
| 3 | exp *Plasmodium/ | 27161 |
| 4 | plasmodi*.ti,ab. | 43158 |
| 5 | or/1-4 | 92461 |
| 6 | exp lipid/ | 1176285 |
| 7 | exp lipid metabolism/ | 218446 |
| 8 | exp "disorders of lipid and lipoprotein metabolism"/ | 230669 |
| 9 | (lipid* or lipoprotein* or apolipoprotein* or chylomicron* or HDL* or IDL or IDLs or LDL* or VLDL* or alphalipoprotein* or betalipoprotein* or phospholipid* or fatty acid* or sterol* or cholesterol* or fats or glyceride* or triglyceride* or lipogenes* or lipolys* or lipoylation* or dyslipidemia* or dyslipoproteinemia* or hyperlipidemia* or lipidemia* or lipemia* or hyperlipoproteinemia* or hypertriglyceridemia* or hypercholesterolemia* or hypercholesteremia* or hypolipoproteinemia* or hypocholesterolemia*).ti,ab. | 864502 |
| 10 | or/6-9 | 1594086 |
| 11 | 5 and 10 | 2874 |
| 12 | (animal* or plant*).hw. not human/ | 4195149 |
| 13 | 11 not 12 | 2101 |
| 14 | limit 13 to (conference abstract or conference paper or conference proceeding or "conference review") | 232 |
| 15 | 13 not 14 | 1869 |

Database: **Cochrane Central Register of Controlled Trials (The Cochrane Library, 10 July 2013)**

Date of search: 10 July 2013

| *#* | *Searches* | *Results* |
| --- | --- | --- |
| #1 | [mh Malaria] or [mh Plasmodium] or (malaria* or plasmodi*):ti,ab,kw | 2903 |
| #2 | [mh Lipids] or [mh "Lipid Metabolism"] or [mh "Lipid Metabolism Disorders"] or (lipid* or lipoprotein* or apolipoprotein* or chylomicron* or HDL* or IDL or IDLs or LDL* or VLDL* or alphalipoprotein* or betalipoprotein* or phospholipid* or fatty acid* or sterol* or cholesterol* or fats or glyceride* or triglyceride* or lipogenes* or lipolys* or lipoylation* or dyslipidemia* or dyslipoproteinemia* or hyperlipidemia* or lipidemia* or lipemia* or hyperlipoproteinemia* or hypertriglyceridemia* or hypercholesterolemia* or hypercholesteremia* or hypolipoproteinemia* or hypocholesterolemia*):ti,ab,kw | 50378 |
| #3 | #1 and #2, in Trials | 38 |

Database: **Web of Science 1975 to July 2013**

Date of search: 11 July 2013

| *#* | *Searches* | *Results* |
| --- | --- | --- |
| #1 | TI=(malaria* OR plasmodi*) | 46526 |
| #2 | TS=(lipid* or lipoprotein* or apolipoprotein* or chylomicron* or HDL* or IDL or IDLs or LDL* or VLDL* or alphalipoprotein* or betalipoprotein* or phospholipid* or "fatty acid*" or sterol* or cholesterol* or fats or glyceride* or triglyceride* or lipogenes* or lipolys* or lipoylation* or dyslipidemia* or dyslipoproteinemia* or hyperlipidemia* or lipidemia* or lipemia* or hyperlipoproteinemia* or hypertriglyceridemia* or hypercholesterolemia* or hypercholesteremia* or hypolipoproteinemia* or hypocholesterolemia*) | 948315 |
| #3 | #1 AND #2 | 942 |
| #4 | #3 NOT (TI=(rodent* OR mouse OR mice OR murine OR rat OR rats OR rabbit* OR cow* OR bovi* OR dog* OR monkey* OR chick* OR fish* OR phyto* OR botan* OR ethnobotan* OR herb* OR fruit*) OR SO=(phyto* OR botan* OR plant* OR veterinary*)) | 847 |

Database: **LILACS (Latin-American and Caribbean Health Sciences Literature) 1982 to July 2013**

Date of search: 11 July 2013

| *#* | *Searches* | *Results* |
| --- | --- | --- |
| 1 | TW:(malaria$ OR plasmodi$) AND TW:(lipid$ OR lipoprotein$ OR apolipoprotein$ OR chylomicron$ OR HDL$ OR IDL OR IDLs OR LDL$ OR VLDL$ OR alphalipoprotein$ OR betalipoprotein$ OR phospholipid$ OR fosfolipid$ OR "fatty acid" OR "fatty acids" OR "acido graso" OR "acidos grasos" OR "acido graxo" OR "acidos graxos" OR sterol$ OR estero$ OR cholesterol$ OR colesterol$ OR fats OR grasas OR gorduras OR glyceride$ OR glicerid$ OR triglyceride$ OR triglicerid$ OR lipogenes$ OR lipolys$ OR lipolis$ OR lipoylation$ OR lipoilac$ OR dyslipidemia$ OR dislipidemia$ OR dyslipoproteinemia$ OR dislipoproteinemia$ or hyperlipidemia$ OR hiperlipidemia$ OR lipidemia$ OR lipemia$ OR hyperlipoproteinemia$ OR hiperlipoproteinemia$ OR hypertriglyceridemia$ OR hipertrigliceridemia$ OR hypercholester$ OR hipercolester$ OR hypolipoproteinemia$ OR hipolipoproteinemia$ OR hypocholesterolemia$ OR hipocolesterolemia$) | 35 |

Database: **BIOSIS Previews 1993 to July 2013**

Date of search: 11 July 2013

| *#* | *Searches* | *Results* |
| --- | --- | --- |
| 1 | (malaria* or plasmodi*).ti,ab. | 41804 |
| 2 | (lipid* or lipoprotein* or apolipoprotein* or chylomicron* or HDL* or IDL or IDLs or LDL* or VLDL* or alphalipoprotein* or betalipoprotein* or phospholipid* or fatty acid* or sterol* or cholesterol* or fats or glyceride* or triglyceride* or lipogenes* or lipolys* or lipoylation* or dyslipidemia* or dyslipoproteinemia* or hyperlipidemia* or lipidemia* or lipemia* or hyperlipoproteinemia* or hypertriglyceridemia* or hypercholesterolemia* or hypercholesteremia* or hypolipoproteinemia* or hypocholesterolemia*).ti,ab. | 501871 |
| 3 | 1 and 2 | 980 |
| 4 | limit 3 to (article or "review articles") | 910 |
| 5 | limit 4 to human | 383 |

Database: **African Index Medicus 1993 to July 2013**

Date of search: 11 July 2013

| *#* | *Searches* | *Results* |
| --- | --- | --- |
| 1 | malaria AND lipid(s), malaria AND lipoprotein(s), malaria AND cholesterol | 0 |
| 2 | Plasmodium AND lipid(s), Plasmodium AND lipoprotein(s), Plasmodium AND cholesterol | 0 |

**Clinical trial registries:**

Search terms used: “Malaria”; “*Plasmodium*” & “plasmodia”

| **Clinical trial registry** | **Total studies identified** | **Registration number(s):** |
| --- | --- | --- |
| Clinicaltrials.gov | 0 | - |
| Controlled-trials.com | 0 | - |
| PROSPERO  http://www.crd.york.ac.uk/NIHR_PROSPERO/ | 0 | - |
| **Total** | 0 | - |

Table B. Search results per clinical trial registry. Search date: 15 July 2013. Updated: 06 November 2013
