# Supplementary material for: Serum lipids and lipoproteins in malaria - a systematic review and meta-analysis
Source: Malar J. 2013 Dec 7;12:442. doi: 10.1186/1475-2875-12-442 (PMC4029227; doi:10.1186/1475-2875-12-442)
Supplement: Additional file 3 — Risk-of-bias assessments. Document with risk of bias assessments for the quality of non-randomized studies included in this meta-analysis. [file 1475-2875-12-442-S3.doc]

**Additional File 3:** Risk-of-bias assessments

**Risk of bias assessment for the quality of nonrandomized studies included in this meta-analysis**

The likelihood that the observations of serum lipid profile changes in malaria reported in this systematic review approximates the truth depends on the validity of the included studies, as certain methodological characteristics may be associated with effect sizes. Therefore, one author (BJV) assessed the validity of the eligible studies (controlled clinical trials, cohort studies, case-control studies, case-series and cross-sectional studies) with design specific criteria. The Newcastle-Ottawa Scale (NOS) was used for assessing the quality of non-randomised studies in meta-analyses, University of Ottawa, Ottawa (2011) (<http://www.ohri.ca/programs/clinical_epidemiology/oxford.asp>). Results of the assessment of risk bias for individual studies are shown in the tables below. Because the main findings are consistent across included studies and the available data is limited, no additional sensitivity analysis have been performed to explore the degree to which the main findings of this systematic review were affected by changes in its methods or in the data used from individual studies (which are listed in table 1). Furthermore, sensitivity analysis or meta-regression of the results has many limitations, including a danger of over-interpretation of presented findings. Nevertheless, reporting the assessment of bias is important as it is part of assessing the strength of the body of evidence.

**Supplementary table:** Risk of bias assessment for studies included in the quantitative synthesis (cross-sectional studies/cohort studies)

| **Reference** | **Selection** | | **Comparability** | **Exposure** | **Overall quality assessment score (of a maximum of 5)** |
| --- | --- | --- | --- | --- | --- |
|  | **Representativeness of the sample** | **Ascertainment of exposure** | **Comparability of the groups on the basis of design or analysis** | **Assessment of outcome** |  |
| Erel et al. 2008 | *Probable representative for the average patient with malaria (*P. vivax*) in Turkey | *Positive malaria slide for *P. vivax* malaria + clinical symptoms | *Controls were available in this study, slide-negative for malaria, healthy study controls for age & sex. | *Independent blind assessment | 4 |
| Kim et al. 2008 | *Truly representative for the average patient with malaria (*P. vivax)*  in Korea | *Patients diagnosed with *P. vivax* malaria (positive blood smear) | **34 non-malaria fever patients, and 52 healthy subjects that were asymptomatic and aparasitemic | *Independent blind assessment | 5 |
| Djoumessi 1989 | Selected group of people complaining of headache and hyperthermia, and of them the people with positive malaria slide. | *Plasmodium detection on thin blood smear | Study does not control for other factors | *Independent blind assessment | 2 |
| Mohanty et al. 1992 | Unknown | *Plasmodium/trophozoites detection on thin blood smear | Study does not control for other factors | *Independent blind assessment | 2 |
| Cuisinier-Raynal et al.1990 | *Somewhat representative of the average patient in the community | *Positive malaria slide for *P. falciparum* | Study does not control for other factors | *Independent blind assessment | 3 |
| Sumitha et al. 1996 | *Truly representative of the average child in the community | *Positive malaria slide for *P. vivax* | Study does not control for other factors | *Independent blind assessment | 3 |
| Njoku et al. 2001 | Selected group of people presenting to hospital with complaints of malaria | Not reported | Controls available, not reported if healthy or symptomatic. | *Independent blind assessment | 1 |
| Agbedana et al. 1990 | *Truly representative of the average child in the community | *Positive malaria slide with *P. falciparum* | *Controls available, both healthy and febrile patients with malaria, however, diagnosis unreported. | *Independent blind assessment | 4 |
| Seshadri et al. 1981 | *Somewhat representative of the average patient in the community | *Microscopical examination of blood smear | Study does not control for other factors | *Independent blind assessment | 3 |
| Das et al. 1996 | Selected group of children, admitted to the ward. Not representative for the average child in the community | *smear positive *P. falciparum* malaria | Study does not control for other factors | *Independent blind assessment | 2 |
| Selvam et al. 1992 | *Somewhat representative of the average patient in the community | *trophozoite stages and gametocyte stages of *P. vivax* in peripheral blood smear | *Controls available, both healthy and febrile patients with malaria, however, age/sex matched | *Independent blind assessment | 4 |
| Mfonkeu et al. 2010 | *Truly representative of the average child in the community | * Blood was spotted on the slide, and thick films were prepared in duplicate. The parasites were counted with a microscope | **Study controls for age, sex, anthropometric measurements | *Independent blind assessment | 5 |
| Eteng et al. 2010 | *Somewhat representative of the average pregnant patient in the community | **Plasmodium falciparum* parasites by microscopic examination of the Giemsa stained blood smear from finger pricks. | *Study controls for age, sex. | * Independent blind assessment | 4 |
| Al Omar et al. 2010 | *Truly representative of the average patient in the community | *Not reported | *Study controls for age, sex. | * Independent blind assessment | 4 |
| Ogbodo et al. 2008 | *Somewhat representative of the average child in the community | *thick blood films examined under the microscope using ×100 objective lens | Study controls for age, not for other factors. | * Independent blind assessment | 3 |

**Supplementary table:** Risk of bias assessment for studies included in the quantitative synthesis (case-control).

| **Reference** | **Selection** | | | | **Comparability** | **Exposure** | | **Overall quality assessment score (of a maximum of 8)** |
| --- | --- | --- | --- | --- | --- | --- | --- | --- |
|  | **Is the case definition adequate?** | **Representativeness of the cases** | **Selection of controls** | **Definition of controls** | **Comparability of cases or controls on the basis of design or analysis** | **Ascertainment of exposure** | **Same method of ascertainment for cases and controls** |  |
| **Faucher et al.2002** | *Yes, with independent validation | *Consecutive or obviously representative series of cases | *Hospital controls | *Adequate, negative for *P. falciparum* at entry of study and no other symptoms | *Study controls for treatment | *Secure record | *Yes | 7 |
| **Parola et al. 2004** | *Yes, with independent validation | *Consecutive or obviously representative series of cases | *56 patients with fever from the tropics but without malaria | n/a | n/a | *Secure record | n/a | 4 |

**CODING MANUAL FOR COHORT STUDIES (NOS)**

# SELECTION

1. **Representativeness of the Exposed Cohort**

Item is assessing the representativeness of exposed individuals in the community, not the representativeness of the sample of women from some general population. For example, subjects derived from groups likely to contain middle class, better educated, health oriented women are likely to be representative of postmenopausal estrogen users while they are not representative of all women (e.g. members of a health maintenance organisation (HMO) will be a representative sample of estrogen users. While the HMO may have an under-representation of ethnic groups, the poor, and poorly educated, these excluded groups are not the predominant users users of estrogen).

Allocation of stars as per rating sheet

1. **Selection of the Non-Exposed Cohort**

Allocation of stars as per rating sheet

1. **Ascertainment of Exposure**

Allocation of stars as per rating sheet

1. **Demonstration That Outcome of Interest Was Not Present at Start of Study**

In the case of mortality studies, outcome of interest is still the presence of a disease/ incident, rather than death. That is to say that a statement of no history of disease or incident earns a star.

***COMPARABILITY***

1. **Comparability of Cohorts on the Basis of the Design or Analysis**

A maximum of 2 stars can be allotted in this category.

Either exposed and non-exposed individuals must be matched in the design and/or confounders must be adjusted for in the analysis. Statements of no differences between groups or that differences were not statistically significant are not sufficient for establishing comparability. Note: If the relative risk for the exposure of interest is adjusted for the confounders listed, then the groups will be considered to be comparable on each variable used in the adjustment.

There may be multiple ratings for this item for different categories of exposure (e.g. ever vs. never, current vs. previous or never)

Age + sex = , Other controlled factors =

***OUTCOME***

1. **Assessment of Outcome**

For some outcomes (e.g. fractured hip), reference to the medical record is sufficient to satisfy the requirement for confirmation of the fracture. This would not be adequate for vertebral fracture outcomes where reference to x-rays would be required.

1. Independent or blind assessment stated in the paper, or confirmation of the outcome by reference to secure records (x-rays, medical records, etc.)
2. Record linkage (e.g. identified through ICD codes on database records)
3. Self-report (i.e. no reference to original medical records or x-rays to confirm the outcome)
4. No description.
5. **Was Follow-Up Long Enough for Outcomes to Occur**

An acceptable length of time should be decided before quality assessment begins (e.g. 5 yrs. for exposure to breast implants).

1. **Adequacy of Follow Up of Cohorts**

This item assesses the follow-up of the exposed and non-exposed cohorts to ensure that losses are not related to either the exposure or the outcome.

Allocation of stars is as per rating sheet.
