# Supplementary material for: Serum lipids and lipoproteins in malaria - a systematic review and meta-analysis
Source: Malar J. 2013 Dec 7;12:442. doi: 10.1186/1475-2875-12-442 (PMC4029227; doi:10.1186/1475-2875-12-442)
Supplement: Additional file 4 — Statistical analysis. The data provided describes the statistical analysis including the inverse-variance methods for combining results across studies (the meta-analysis), calculation of pooled mean and standard deviation and rounding of the data. [file 1475-2875-12-442-S4.doc]

**Additional File 4:** Statistical Analysis

**Title:** Serum lipids and lipoproteins in malaria – a systematic review and meta-analysis.

**Authors:** Benjamin J. Visser; Rosanne. W. Wieten; Ingeborg M. Nagel; Martin P. Grobusch

**Date:** 09 September 2013

**Version:** 1.0

**Meta-analysis method:**

**Inverse-variance methods for combining results across studies¹**

Inverse-variance methods are used to pool log odds ratios, log risk ratios and risk differences as one of the analysis options for binary data, to pool all mean differences and standardized mean differences for continuous data, and also for combining intervention effect estimates in the generic method. In the general formula the intervention effect estimate is denoted by
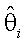
, which is the study’s log odds ratio, log risk ratio, risk difference, mean difference or standardized mean difference, or the estimate of intervention effect in the generic method. The individual effect sizes are weighted according to the reciprocal of their variance (calculated as the square of the standard error given in the individual study section above) giving


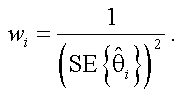


These are combined to give a summary estimate


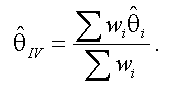


With


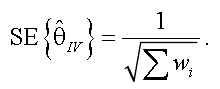


The heterogeneity statistic is given by a similar formula as for the Mantel-Haenszel method:


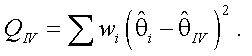


Under the null hypothesis that there are no differences in intervention effect among studies this follows a chi-squared distribution with k-1 degrees of freedom (where is the number of studies contributing to the meta-analysis). I² is calculated as


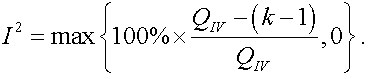


**To calculate the pooled mean and pooled standard deviation²:**

For four records (Mohanty et al. 1992; Das et al. 1996; Njoku et al. 2001, Mfonkeu et al. 2010) in our meta-analysis, we wanted to combine two series of measurements performed under similar conditions in the same patient group (=malaria patients) to achieve an improved estimate of the imprecision of the process. If it can be assumed that all the series in one study are of the same precision although their means may differ, the pooled standard deviations
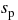
from
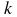
series of measurements can be calculated as


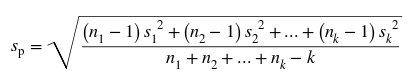


The suffices 1 , 2 , ... , *
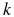
* refer to the different series of measurements. In this case it is assumed that there exists a single underlying standard deviation
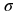
 of which the pooled standard deviation
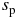
 is a better estimate than the individual calculated standard deviations
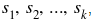
For the special case where
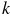
 sets of duplicate measurements are available, the above equation reduces to


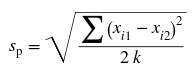


Results from various series of measurements can be combined in the following way to give a pooled relative standard deviation
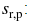


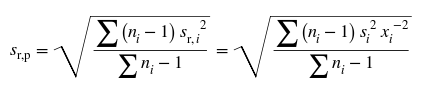


Online calculator available at:

<http://home.ubalt.edu/ntsbarsh/Business-stat/otherapplets/Pooled.htm>

(Courtesy of Prof. Hossein Arsham) – Last accessed 10 September 2013

**Rounding of data³:**

For the data entry in Review Manager we used the round-to-nearest method (to two decimal places). This is a form of unbiased rounding. For example: 3.64754 becomes 3.65 or 3.64454 becomes 3.64.

***References:***

1. *Adapted and modified from: Deeks JJ, Higgins JPT on behalf of the Statistical Methods Group of The Cochrane Collaboration. Statistical algorithms in Review Manager 5. August 2010.*
2. *IUPAC. Compendium of Chemical Terminology, 2nd ed. (the "Gold Book"). Compiled by A. D. McNaught and A. Wilkinson. Blackwell Scientific Publications, Oxford (1997). XML on-line corrected version: http://goldbook.iupac.org (2006-) created by M. Nic, J. Jirat, B. Kosata; updates compiled by A. Jenkins. ISBN 0-9678550-9-8. doi:10.1351/goldbook.*
3. *[No authors listed]. Rounding; Round half to even. http://en.wikipedia.org/wiki/Rounding (last accessed 10 September 2013).*
