# Supplementary material for: Serum lipids and lipoproteins in malaria - a systematic review and meta-analysis
Source: Malar J. 2013 Dec 7;12:442. doi: 10.1186/1475-2875-12-442 (PMC4029227; doi:10.1186/1475-2875-12-442)
Supplement: Additional file 5 — Stratified meta-analysis for uncomplicated and severe malaria. Document providing results of the meta-analysis (total cholesterol) stratified for uncomplicated malaria and severe malaria. [file 1475-2875-12-442-S5.doc]

**Additional file 5:**

**Stratified meta-analysis for uncomplicated and severe malaria*.**

*Definitions of uncomplicated and severe malaria adapted from the included studies. For all studies, differences between serum cholesterol levels between malaria cases and healthy controls are given; three studies provided information on the degree of severity and were included into the meta-analysis.

Forest plot. Mean difference for cholesterol (mmol/l) between ***severe*** malaria patients and healthy controls. Random-effect model. Mean difference: 1.60 mmol/l or 61.87 mg/dl (95% CI 0.66-2.54), I²=99%, Z=3.33, P= 0.0009

Forest plot. Mean difference for cholesterol (mmol/l) between ***uncomplicated*** malaria patients and healthy controls. Random-effect model. Mean difference 1.01 mmol/l or 39.06 mg/dl (95% CI 0.63-1.39), I²=99%, Z=5.22, P<0.00001
