# Supplementary material for: Serum lipids and lipoproteins in malaria - a systematic review and meta-analysis
Source: Malar J. 2013 Dec 7;12:442. doi: 10.1186/1475-2875-12-442 (PMC4029227; doi:10.1186/1475-2875-12-442)
Supplement: Additional file 6 — Forest plots & funnel plots. The data provided describes the full results of the meta-analysis for the different lipid parameters investigated. To increase transparency, both fixed and random effect analyzes are shown in this document. [file 1475-2875-12-442-S6.doc]

**Additional File 6:** Forest plots & funnel plots.

(All values are in mmol/l. Both fixed as well as random models are shown)

Malaria vs. healthy controls. Outcome: cholesterol. Fixed effect model.

Malaria vs. healthy controls. Outcome: cholesterol. Random effect model.

Funnel plot. Malaria vs. healthy controls. Outcome: cholesterol. Random effect model.

Malaria vs. symptomatic controls. Outcome: cholesterol. Fixed effect model.

Malaria vs. symptomatic controls. Outcome: cholesterol. Random effect model.

Funnel plot malaria vs. symptomatic controls outcome cholesterol. Random effect model.

Malaria vs. healthy controls, outcome: HDL. Fixed effect model.

Malaria vs. healthy controls. Outcome: HDL. Random effect model.

Funnel plot. Malaria vs. healthy controls. Outcome: HDL.

Malaria vs. symptomatic controls. Outcome: HDL. Fixed effect model.

Malaria vs. symptomatic controls. Outcome: HDL. Random effect model.

Malaria vs. healthy controls. Outcome: LDL. Fixed effect model.

Malaria vs. healthy controls. Outcome: LDL. Random effect model.

Funnel plot. Malaria vs. healthy controls. Outcome: LDL.

Malaria vs. healthy controls. Outcome: LDL. Random effect model. Scale adjusted (2.0 instead of 4.0).

Malaria vs. symptomatic controls. Outcome: LDL. Fixed effect model.

Malaria vs. symptomatic controls. Outcome: LDL. Random effect model.

Malaria vs. healthy controls. Outcome: Triglycerides. Fixed effect model.

Malaria vs. health controls. Outcome: triglycerides. Random effect model.

Funnel plot. Malaria vs. healthy controls. Outcome: triglycerides.

Malaria vs. healthy controls. Outcome: triglycerides. Random effect model. Scale adjusted (2.0 instead of 4.0).

Malaria vs. symptomatic controls. Outcome: triglycerides. Fixed effect model.

Malaria vs. symptomatic controls. Outcome: triglycerides. Random effect model.

**Review Manager 5**

Properties:

Graph Size: 4.00

Sort: year of publication

[parameter] Higher

[parameter] Lower

Malaria cases

Healthy controls/ symptomatic controls
