# Supplementary material for: Serum lipids and lipoproteins in malaria - a systematic review and meta-analysis
Source: Malar J. 2013 Dec 7;12:442. doi: 10.1186/1475-2875-12-442 (PMC4029227; doi:10.1186/1475-2875-12-442)
Supplement: Additional file 7: Table S1 — Studies included in the systematic review and meta-analysis. [file 1475-2875-12-442-S7.doc]

| **Source (first author, year of publication)**  **(PubMed ID)**  ***Language*** | **Country (study site)** | **Study-design + Day of sample collection** | **No. of participants with malaria** | **No. of controls** | **Measure of outcome** | **Total cholesterol (TC)** | **High-density lipoprotein (HDL)** | **Low-density lipoprotein (LDL)** | **Triglycerides (TG)** | **Other findings** |
| --- | --- | --- | --- | --- | --- | --- | --- | --- | --- | --- |
| *Badiaga et al. 2002*  *(12088575)*  *English* | France | Retrospective case-control in febrile patients returning from the tropics. Day: “0”  Mean age* (SD) pt.  34 ± 11 y.  Controls: 36 ± 14 y. and male sex* (67.5% vs. 67.4%).  *NS | 129 total  116 *P. f*  6 *P. o*  5 *P. v*  2 *P. m* | 92 total  24 viral disease  55 bacterial disease  8 parasit disease  5 non-infectious | Odds Ratio + frequency (%) | Hypocholesterolemia  40% (21/53) patients  2% (1/45) controls  Adjusted OR 75.22 (95% CI 4.6-1227.78)  P<0.05  Hypocholesterolemia more frequent in infection with *P. falciparum* (20/26) than in other species (1/7). P-value: NR  Cutt off: < 2,39 g/dl  **Lowered** | NR | NR | Hypertriglyceridemia  56% (28/53) patients  33% (15/45) controls  OR  P<0.05  Cut off: > 1.54 g/L  **Raised** | -Sensitivity, specificity, positive predictive value of hypo-cholesterolemia for malaria respectively 40% (CI -); 98% (CI 95% 0.95-1) and 96%(CI -)  -Among cases, there was no significant differences between African natives and French natives for frequency of hypocholesterolemia (12/24 vs. 16/29, p=0.92) |
| *Chagnon et al. 1993*  *(8191080)*  *French* | France | Cross-sectional  Day: NR | 64 total  52 *P. f.*  12 non-falciparum | n/a | Frequency (%) | Hypocholesterolemia45% ( 29/64 patients low cholesterol)  Cut off: 1 g/L  **Lowered** | 80% (51/64 patients low HDL)  Cut off: NR  **Lowered** | NR | 42,3% (27/64 patients high triglycerides)  Cut off: 2g/l  **Raised** | - |
| *Chukwuocha et al. 2011*  *(22118037)*  *English* | Nigeria | Prospective observational cohort in patients with “clinical signs”  Day: “0” | 55 total *P. f.* | 55 controls related in age and sex (patients with low parasite count 1-10 per 100 high power field) | Frequency (%), Range | Hypocholesterolemia  84% (46/55 patients) with low cholesterol, 1/55 normal, 5/55 raised.  6% (3/55) controls  P-value: NR  [Range] patients: [168-274] vs. [138-220] Means: NR  Cut off: <180mg/dL  **Lowered** | NR | NR | NR | - a significant correlation (12.9%, P<0.01) between malaria parasite status and cholesterol level. |
| *Davis et al. 1993*    *(8505562)*  *English* | Australia | Prospective observational cohort study in Chinese rural workers  Day: “0” | 18 total  10 *P. f.*  8 *P. v.* | 10 healthy controls | Frequency (%) Medians and ranges | Medians mmol/l [range]  *P. f* 2.1 [1.2-4.3]*  *P. v.* 2.5 [1.5-4.0]*  Controls 4.7 [ 3.4-6.6]  *P <0,001 vs. controls  100% (18/18 low cholesterol)  **Lowered** | Medians mmol/l [range]  *P. f.*  0.4 [0.1I-0.9]*  *P. v.*. 0.4 [0.2-0.8]*  Controls: 1.4 [1.1-1.9]  *P< 0,001 vs. controls  100% (18/18 low HDL)  **Lowered** | Medians mmol/l [range]  *P. f.*  1.0 [0.1-2.8]*  *P. v. 1*.6 *[0.8-2.2]  Controls 2.6 [2.3-4.9]  *P<0.01 vs. controls  100% (18/18 low LDL)  **Lowered** | Medians mmol/l [range]  *P. f.* 1.4 [0.4-4.4]*  *P. v.* 1.1 [0.6-2.7]*  Controls 1.0 [0.3-2.4]  *P 0,036  Numbers NR  **Raised** | -No significant relation between total serum cholesterol and parasitemia  -Values in subgroups (*vivax* vs*. falciparum*.) not significant (P 0.18)  -HDL/Total cholesterol ratio lowered in *P. v/f* |
| *Erel et al. 1998*  *(10069446)*  *English* | Turkey | Prospective observational cohort study  Day: “0”, after overnight fasting | 60 patients  60 *P. v.* | 50 healthy age and sex matched controls | Means and standard deviation | Means mmol/l, SD, P-value  *P. v.* 2.96, (0.80),  P= 0.001 vs. controls  Controls 3.40 (1.12)  **Lowered** | Means mmol/l, SD, P-value  *P .v.* 0.37 (0.28) P<0.001 vs. controls  Controls 1.10 (0.79)  **Lowered** | Means mmol/l, SD, P-value  *P. v.* 1.45 (0.82)  P=0,005 vs. controls  Controls 2.29 (1.00)  **Lowered** | Means mmol/l, SD, P-value  P.v. 2.19 (0.99)  P=0.01 vs. controls  Controls 1.65 (0.93)  **Raised** | Plasma lipid peroxidation (TBARS) (umol/l)  *P. v.* 1.33 (0.23)  P<0.001 vs. controls  Controls 0.81 (0.26) |
| *Faucher et al. 2002*  *(12444452)*  *English* | Gabon | Retrospective selected samples from a malaria profylaxis study  Day “0” and “35” | 47 total  *P. f.*  47 (all with low parasitemia, below 1000/ul parasites) | 47 healthy controls  Note: this study compares within the patient & control group (D0 vs. D35), not between the patient and control groups! | Means and standard deviation | Mean mmol/l (SD) P-value (D0 vs. D35)  *47 P. f.* D0: 3.49 (0.88) D35: 3.99 (0.70) P<0.001  sig. increase between D0 & D35  47 controls D0: 3.87 (0.66) D35:3.90 (0.63) P0.60  -no sig. diff. between D0 & D35  -*21 P. f.* with very low parasitemia (VLP) (<100/ul)  D0: 3.60 (0.90) D35:3.86 (0.80) P0.03  -sig. diff between D0 & D35  **Lowered** | Mean mmol/l (SD) P-value (D0 vs. D35)  *47 P. f.*D0: 0.63 (0.25) D35: 0.91 (0.24) P<0.001  - sig. increase between D0 & D35  47 controls: D0: 1.01 (0.35) D35 1.08 (0.29) P0.05  -no sig. diff. between D0 & D35  -*21 P. f.* with very low parasitemia *(VLP) (<100/ul)*  D0: 0.58 (0.35) D35: 0.83 (0.29) P<0.001  -sig. diff. Between D0 & D35  **Lowered** | Mean mmol/l (SD) P-value (D0 vs. D35)  *47 P. f.* D0: 1.61 (0.65) D35: 1.65 (0.43) P0.93  -no sig. diff. between D0 & D35  47 controls: D0: 1.71 (0.61) D35: 1.57 (0.57) P0.17  -no sig. diff. between D0 & D35  -*21 P. f.* with very low parasitemia *(VLP) (<100/ul)*  D0: 1.64 (0.68) D35: 1.66 (0.52) P0.96  -no sig. diff. between D0 & d35  **Lowered** | Mean mmol/l (SD) P-value (D0 vs. D35)  *47 P. f.* D0: 0.93 (0.35) D35: 0.77 (0.24) P0.005  -sig. diff. between D0 & D35  47 controls: DO 0.74 (0.40) D35: 0.67 (0.21) P0.26  - no. sig. diff. between D0 & D35  -*21 P. f.* with very low parasitemia *(VLP) (<100/ul)*  D0: 0.90 (0.33) D35: 0.80 (0.29) P0.08  -no sig. diff between D0 & D35  **Raised** | -sig. lipid changes related  to low-level P. falciparum infections  -After parasite clearance, mean values were  significantly increased for Tchol (P<0.001) and HDL-c  (P<0.001), unlike LDL-c (P=0.93); and TG were significantly  decreased from D0 (P=0.005). No sig. change was found in a control group. |
| *Grobusch et al. 2003*  *(12718813)*  *English* | Germany | Case report of *P.* *falciparum* in patient with Tangier disease  Day: NR, D28 | 1 *P. falciparum* patient | - | Individual values | 27mg/dL  (0.69822 mmol/l  Ref. value: NR  **Lowered** | 3mg/dL  0.07758 mmol/l  Ref. value: NR  **Lowered** | NR | 231mg/dL  2.60799 mmol/l  Ref. value: NR  **Raised** | -Apolipoprotein A1 below detecton levels. -Persisting abnormal lipids level on D28. |
| *Kim et al. 2008*  *(18304498)*  *English* | Korea | Prospective observational cohort study of patients presenting with fever  Day: “0”, D3, D17, D28, Month 6, M12 | 55 patients  55 *P. vivax* | Two control groups:  Patients with fever (n=34)  Health control (n=52) | Means and standard deviation | Data presented for Day: “0” Mean mg/dL (SD), P-Value  *P. v.* 76.1 (27.1) mg/dl  1.96795 (0.70081) mmol/l P<0.01  Fever pt: 130.5 (26.3) mg/dl 3.37473 (0.68012) mmol/l  Healthy-controls: 169.5 (25.8) mg/dl  4.38327 (0.66719) mmol/l  Ref. Value 150-240  **Lowered** | Data presented for Day: “0” Mean mg/dL (SD), P-Value  *P. v.* 16.7 (8.9) mg/dl  0.43186 (0.23015) mmol/lP<0.01  Fever pt: 38.1 (19.0) 0.98527 (0.49134) mmol/l  Healthy controls: 57.3 (12.5)  1.48178 (0.32325) mmol/l  Ref. Value 30-85  **Lowered** | Data presented for Day: “0” Mean mg/dL (SD), P-Value  *P. v*. 29.6 (15.5) 0.76546 (0.40083) mmol/lP<0.01  Fever pt: 66.6 (26.2) 1.72228 (0.67753) mmol/l  Healthy controls 94.3 (24.1)  2.4386 (0.62323) mmol/l  Ref. Value 65-160  **Lowered** | Data presented for Day: “0” Mean mg/dL (SD), P-Value  P. v. 95.9 (59.2) 1.08271 (0.66837) mmol/l P >0.05  Fever pt: 101.3 (81.2) 1.14368 (0.91675) mmol/l  Healthy- controls 98.2 (63.7)  1.10868 (0.71917) mmol/l Ref 50-200  **No sig. difference** | -Lipid parameters of patients resolved slowly; after 1 & 6 months lipid levels were still increased, HDL-c and LDL-c returned to normal at month 12 |
| *Lambrecht et al. 1978*  *(77973)*  *English* | Belgium | Consecutive Case-series in patients with P. *vivax* returning from the tropics  Day: NR | 6 patients with *P. vivax* | - | Frequency (%) | NR | 6/6 cases no HDL -could be detected using cellulose-acetate electrophoresis  **Lowered** | LDL-c: NR  3/6 cases no very low-density lipoprotein (VLDL) could be detected.  **VLDL Lowered** | NR | - |
| *Ngou-Milama et al. 1995*  *(7780673)*  *French* | Gabon | Prospective observational study in Gabonese *children* 18 months-4 y o treated with halofantrine.  Day: 0, 7 & 14 | 51 patients with malaria, sub-species not specified | - | Means and standard deviation | Data presented for Day:”0” Mean mmol/l (SD), P-value  Patients: 2.35 (0.70) P= “significant”  Reference value: 1.80 (0.98)  **Raised** | Patients: 0.45 (0.35) P= “significant”  Reference value: 1.34 (0.26)  **Lowered** | LDL > NR  -Non-esterified fatty acids (NEFAs):  Patients: 1.10 (0.50) P = “significant”  Reference value: 0.60 (0.35) | Patients: 2.15 (1.00)  P= “significant”  Reference value: 0.55 (0.40)  **Raised** | -Total cholesterol and HDL-c raised during treatment (D14 still abnormal), triglycerides declined to normal on D14 |
| *Parola et al. 2004*  *(14963768)*  *English* | France | Retrospective case-control study patients with fever returning from tropics with malaria  Day: “0” | 278 patients  222 malaria pt  *P. f* 198 (89%) (183 pt mild, 14 pt severe)  *P. v* 14 (6%)  *P. o* 8 (4%)  *P. m* 2(2%) | 56 patients with fever from the tropics but without malaria | Means, ranges and standard deviation | NR | NR | NR | (Patients tested) Mean mmol/l (SD) [Range] P-value  (13) *P. f.* (severe) 4.78 (1.93) [2.3-10.1]*  (169) *P. f.* (mild) 1.94 (1.11) [0.5-5.8) *  (20) Non*-falciparum* 1.92 (1.14) [0.5-4.1) *  *P-value <0.05  Ref value: 0.4-1.8 mmol/l  Malaria patients 2.17 (1.43) [0.5-10.1]  Controls 1.30 (0.70) [0.5-3.3]  **Raised** | In severe malaria, 100% of the patients had hypertriglyceridemia, but only 37% had hypertriglyceridemia in the mild falciparum group (P<0.001). |
| *Nilsson-Ehle et al. 1990*    *(2179459)*  *English* | Sweden | Prospective observational study in patients returning from the tropics with malaria: Day: “0”, D2-4, D7, D14.  Mean age: 32 y (18-49) | 16 malaria pt  7 *P. falciarum*  7 *P. vivax*  1 *P. ovale*  1 *P. falciparum & ovale*  Rx : chloroquine and/or primaquine | - | Frequency (%) Means and range | NR | 16 patients (100%) with low HDL. Day 0:  Mean: 0.19 mmol/l Range 0.06-0.5 mmol/l  Ref value 0.8-1.6 mmol/l  HDL levels normalized after 2-3 weeks.  **Lowered** | 16 patients (100%) with low LDL. Day 0:  Mean: 1.01 mmol/l Range: 0.43-1.8mmol/l  Ref value 2.2-5.2 mmol/l  LDL normalized after 2 weeks.  **Lowered** | 16 patients (100%) with hypertriglyceridemia Day 0:  Mean: 2.31 mmol/l  Range 1.2-3.32 mmol/l  Ref value: 0.7-2.2  TG normalized after 2 weeks.  **Raised** | -Low HDL-c levels were related to parasitemia  -no significant differences found between patterns from different infections with *P. ovale, P. falciparum, P. vivax*. |
| *Kittl et al. 1992*  *(1546481)*  *German* | Germany | Prospective observational cohort study. Day: “0” | Total 104 patients  68 malaria patients  41 *P. falciparum (“tropica”)* (10 patients with severe malaria)  23 *P. ovale/ vivax (“tertiana”)*  4 mixed infection *P. f & P. v* | 23 patients with tropical infections (Typhoid fever, Rickettsiosis and dengue)  13 healthy controls | Frequency (%), Median, range | %, median mg/dl, [range]  68 malaria pt  50 patient(73%) with low TC  Median: 104 mg/dl [56-256]  2.68944 (1.44816- 6.62016) mmol/l  (Ref value 140-200 mg/dl)  23 controls with tropical infections: Median: 139 mg/dl [84-289 mg/dl] 3.59454 (2.17224- 7.47354) mmol/l  **Lowered** | %; median mg/dl, [range]  68 malaria pt  46 ♂, 22 ♀  42 ♂malaria pt (91%) low HDL Median 9.5 mg/dl [3-48](Ref value: 35-55)  0.24567 (0.9051-1.4223) mmol/l  20 ♀ (91%) low HDL Median: 14.5 mg/dl [2-54] 0.37497 (0.05172- 1.39644) mmol/l (Ref value: 45-65)  23 controls with tropical infections: Median: 29 mg/dl [12-48] 0.74994 (0.31032- 1.24128) mmol/l  **Lowered** | NR | %; median mg/dl, [range]  68 malaria pt  43 (63%) high TG;  Median 211 mg/dl [37-725] (Ref value <170mg/dl)  2.38219 (0.41773- 8.18525) mmol/l  23 controls with tropical infections: Median: 156 mg/dl [56-462]  1.76124 (0.63224- 5.21598) mmol/l  **Raised** | -Extent of HDL decrease no correlation with disease severity  -HDL-c as independent diagnostic marker for malaria  -In controls (with tropical infections), different pattern of lipid profile changes  -Apolipoprotein A and A-1 lowered in malaria patients, correlation with HDL-c very strong (r=0.96 for Apo A & r= 0.95 for Apo A-1)  -follow up in “a low number of patients” showed normalized HDL after 10-15 days. |
| *Baptista et al. 1996*  *(9033909)*  *French* | São Tomé | Cross-sectional observational study in children aged 4-14 years old presenting with fever.  Day: “0”  Rx: quinine | Total 88 patients  2 excluded  44 uncomplicated malaria (UCM)  12 severe malaria (SM) | 30 healthy controls matched for age & sex | Median mg/dl [range], p-value | Median mg/dl, [range], p-value  44 UCM patients Median: 90.5 mg/dl [72-115 mg/dl]  2.34033 [1.86192- 2.9739] mmol/l  12 SM patients Median: 108.5 [82-129.5 mg/dl]  2.80581 [2.12052- 3.34887] mmol/l  30 healthy controls Median: 124.5 [108-146] 3.21957 [2.79288- 3.77556] mmol/l  Ref value 112-248 mg/dl  P-value: <0.001 (controls vs. UCM) P-value NS for controls vs. SM or UCM vs. SM)  **Lowered** | Median mg/dl [range], p-value  44 UCM patients Median: 1 mg/dl [0-10 mg/dl]  0.02586 [0- 0.2586] mmol/l  12 SM patients Median: 0 mg/dl [0-14.5 mg/dl]  0 [0- 0.37497] mmol/l  30 healthy controls Median: 32.5 mg/dl [27-39 mg/dl]  0.84045 [0.69822- 1.00854] mmol/l  Ref value: 36-84 mg/dl  P-value:<0.001 (controls vs. UCM), <0.001 (controls vs. SM) and NS (UCM vs. SM)  **Lowered** | Median mg/dl [range], p-value  44 UCM patients  Median: 55.7 mg/dl [38.9-73.6 mg/dl]  1.4404 [1.00595- 1.9033] mmol/l  12 SM patients Median: 57 mg/dl [35.1-80.9]  1.47402 [0.90769- 2.09207] mmol/l  30 healthy controls Medan: 76.9 mg/dl [61.8-103.4]  1.98863 [1.59815- 2.67392] mmol/l  Ref value: ≤ 130 mg/dl  P-value: <0.01 (controls vs. UCM), P-value NS (controls vs. SM or UCM vs. SM)  **Lowered** | Median mg/dl [range], p-value  44 UCM patients  Median: 135 mg/dl [91-172.5]  1.52415 [1.02739- 1.94753] mmol/l  12 SM patients Median: 144.5 mg/dl [ 89.5-289 mg/dl]  1.63141 [1.01045- 3.26281] mmol/l  30 healthy controls  Median: 75 mg/dl [59-91 mg/dl]  0.84675 [0.66611- 1.02739] mmol/l  Ref value: 24-140 mg/dl  P-value: <0.001 (controls vs. UCM), <0.001 (controls vs. SM), NS (UCM vs. SM)  **Raised** | - No correlation between lipid plasma levels and disease severity was found. |
| *Djoumessi 1989*  *(2694079)*  *English* | Cameroon | Cross-sectional observational study. Of 120 patients complaining of headache and hyperthermia, 37 were malaria positive.  Day: NR/”after an overnight fast” | 37 *P. falciparum* patients | 37 healthy controls (student population) | Means , standard deviation and p-value | Means (SD), P-value  37 malaria patients: 1.23 g/l (0.37)  3.18078 (0.95682 ) mmol/l  37 healthy controls 1.61 g/l (0.60)  4.16346 (1.5516 ) mmol/l  P<0.05  **Lowered** | Means (SD), P-value  37 malaria patients: 0.46 (0.36)  1.18956 (0.93096 ) mmol/l  37 healthy controls 0.62 (0.25)  1.60332 (0.6465 ) mmol/l  P<0.05  **Lowered** | Means (SD), P-value  37 malaria patients: 0.77 (0.85)  1.99122 (0.961455 ) mmol/l  37 healthy controls: 0.99 ( 0.33)  2.56014 (0.85338) mmol/l  P<0.05  **Lowered** | NR | Apolipoprotein B concentration higher (P<0.05) in malaria patients  Mean (SD) Patients 1.64 (0.50), controls: 1.03 (0.50) and  a decrease in apolipoprotein A. Mean (SD)  Patients: 1.36 (0.60), controls 1.70 (0.34) suggesting atherogenic risk  -no correlation between parasitemia and TC, HDL, LDL and apolipoproteins (r = 0.39) |
| *Mohanty et al. 1992*  *(1304701)*  *English* | India | Prospective observational study in 60 patients with *P. falciparum* malaria. Day:”0” for all participants, D3, D7, D11, D15, D21 for 22 severe malaria patients. | 60 patients  37 *P. f* malaria (severe)  23 *P. f.* malaria (mild)  Rx. i.v. quinine for severe cases, mild cases with chloroquine | 83 healthy controls (asymptomatic) | Means and standard deviation | Mean (mmol/l), (SD)  23 UCM Mean: 3.32 mmol/l (1.10)*  37 SM Mean: 2.32 mmol/l (0.73)*  83 controls Mean: 4.77 mmol/l (0.91)  Weighted mean malaria (60 patients): 2.70333 (0.87034)  *vs. controls, P-value <0.05, ANOVA: P<0.001  Ref value: NR  After 3 weeks (22 SM patients) TC remained far below those in controls (3.14 mmol/l (0.71) vs. 4.77 mmol/l (0.91)  **Lowered** | Mean (mmol/l), (SD)  23 UCM Mean: 0.773 mmol/l (0.215)*  37 SM Mean: 0.428 mmol/l (0.301)*  83 controls Mean: 1.167 mmol/l (0.170)  Weighted mean malaria 0.56025 mmol/l (0.26837)  *vs. controls, P-value <0.05, ANOVA: P<0.001  Ref value: NR  **Lowered** | Mean (mmol/l), (SD)  23 UCM Mean: 1.663 mmol/l (1.027)*  37 SM Mean: 0.735 mmol/l (0.600)*  83 controls Mean: 2.828 mmol/l (0.802)  Weighted mean malaria 1.09073 (0.76196)  *vs. controls, P-value <0.05, ANOVA: P<0.001  Ref value: NR  **Lowered** | Mean (mmol/l), (SD)  23 UCM Mean: 1.72 mmol/l (0.57)*  37 SM Mean: 2.53 mmol/l (1.29)*  83 controls Mean: 1.69 mmol/l (0.71)  Total weighted mean malaria : 2.2195 (1.01689)  *vs. controls, P-value <0.05, ANOVA P<0.001  Ref value: NR  After 3 weeks (22 patients:  22: patients: 1.72 mmol/l (0.60) vs. 1.69 mmol/l (0.70) in controls. > TG normalized after 3 weeks.  **Raised** | - levels of L particles in the patients with severe malaria were significantly elevated compared with the other patients and controls (P < 0.001), indicating impaired metabolism of chylomicrons.  -albumin, was reduced significantly and was directly correlated to HDL cholesterol levels (r = 0.715 and r = 0.895, respectively) in both mild and severe malaria.  -The estimated levels of S particles, corresponding to LDL, were lower in all malaria cases than in the controls (P < 0.001) |
| *Vernes et al. 1980*  *(6775271)*  *French* | France | Cross-sectional study in travellers returning from the tropics  Day: “0” and “after treatment” | Total 12 patients  7 *P. falciparum*  3 *P. vivax*  1 *P. ovale*  1 mixed infection (*P. falciparum & P. vivax)* | - | Individual values for TC & TG. “+” or “-“ classification for HDL, LDL and VLDL.  NB: Only individual values are available, means could not be calculated. | In 9 of 9 patients (100%) total cholesterol was lowered compared to reference values and after treatment. NB: in 3 patients, TC levels were not determined  Ref values: NR  **Lowered** | In 10 of 10 patients (100%) HDL was very low (n=6) or undetectable (n=4). NB: in 2 patients, HDL values were not measured  Ref values: NR  **Lowered** | In 7 of 10 patients (70%) LDL was lowered.( In 2 patients normal, in 2 patients raised and in 2 not determined.  Ref values: NR  In 9 of 10 patients (90%) VLDL was raised, 1 normal and 2 not determined  Ref values: NR  **LDL Lowered**  **VLDL Raised** | In 9 of 9 patients (100%) triglyceride level was raised.  In 3 patients, TG was not measured. These TG changes normalized after treatment.  Ref values: NR  **Raised** | -alterations in lipid parameters were not correlated with the intensity of parasitaemia or the species of Plasmodium  -all lipid profiles changes returned to normal in a average of 21 days |
| *Cuisinier-Raynal et al. 1990*  *(2366654)*  *French* | France | Cross-sectional observational study with 144 soldiers | Total 144 non-immune patients:  67 *P. f.*  patients  51 health controls  Day: “0” | 51 healthy controls (non febrile), same exposition | Mean (g/l; mmol/l), variance, minimum-maximum value. | Mean (g/l), (variance), min-max. P-value  67 *P. f.* patients: 4.8 (1.04), 2.02-6.96,  12.4128 (2.68944) 5.22372-17.99856 mmol/l  51 healthy controls: 5 (0.89), 2.88-8.15 12.93 (2.30154) , 7.44768-21.0759 mmol/l  P-value: NR/ “not significant”  **No sig. difference** | % of lipoprotein profile (variance), min-max  67 *P. f.* patients: 32.25 (9.98), 8.33-50.12  83.3985 (25.8083) [21.54138-129.61032] mmol/l  51 healthy controls: 38.4 (11.88), 4.43-66.9  99.3024 (30.72168) mmol/l [11.45598-173.0034]  P-value: NR  **No sig. difference** | % of lipoprotein profile (variance), min-max  67 *P. f.* patients: 47.87 (9), 20.55-75.29  123.79182 (23.274) mmol/l  51 health controls: 44.5 (10.76), 14.39-65.58  115.077 (27.82536) mmol/l  P-value: NR  **No sig. difference** | Mean (mmol/l), (variance), min-max, P-value  67  *P. f.* patients: 1.32 (0.57), 0.67-3.06  51 healthy controls: 1.07 (0.57), 0.46-3.26  P-value: NR  **No sig. difference** | -results suggests a dysglobulinemia “more significant “in the malaria infected group  -no significant differences found for VLDL, Apo A1, Apo A2 or Apo B between patients and controls. |
| *Onongbu et al. 1983*  *(6356530)*  *English* | Nigeria | Prospective observational study to study serum lipids during and after treatment | Total 18 patients  18 *P. falciparum*  Day: “0” and 2 weeks after treatment.  9 “moderate infection”  6 “severe infection”  3 unknown severity | - | Mean (SD), p-value | Mean mmol/l (SD), p-value  9 moderate inf. malaria patients during infection Mean: 3.74 (0.62) vs. Mean 3.82 (0.41) after infection. P-value >0.05  6 severe infected malaria patients during infection Mean: 3.47 (0.54) vs. Mean 3.67 (0.43) after infection. P-value > 0.05  **No sig. difference** | NR | NR | Mean mmol/l (SD), p-value  9 moderate inf. malaria patients during infection Mean: 1.88 (0.12) vs. Mean 1.02 (0.08) after infection. P-value <0.01  6 severe infected malaria patients during infection Mean: 2.66 (0.18) vs. Mean 0.95 (0.05) after infection. P-value <0.001  **Raised** | -phospholipid levels were higher during infection than after infection and these increases were stat. sig. (p<0.001 for severe infection and p<0.01 for moderate infection) |
| *Sumitha et al. 1996*  *(9014396)*  *English* | India | Cross-sectional design to study the effect of repeated *P. vivax* malaria infections on the lipid status  Day: “0” | Total 65 patients  “F”: 15 *P. vivax* patients  “F1” 20 *P. v.* pt with 2-3 attacks in 3 months  “F2” 15 *P. v.* pt. 4-5 attacks in 5-6 months  “F3”: 15 *P. v.* pt. with >5 attacks a year | 20 healthy age and sex-matched controls | Mean (SD), p-value | Mean mg/dl (SD), p-value  F: 211.31 mg/dl (19.49)*  5.46448 (0.50401) mmol/l  F1: 154.89 mg/dl (7.83) *  F2: 120.18 mg/dl (8.44) *  F3: 109.67 mg/dl (9.13) *  C: 235.68 mg/dl (26.31) *  6.09468 (0.68038) mmol/l  *P-value< 0.001 (compared to control, fresh malaria and repeated malaria)  **Lowered** | NR | NR | Mean mg/dl (SD), p-value  F: 138.39 mg/dl (15.40)*  1.56242 (0.17387) mmol/l  F1: 147.76 mg/dl (16.23) *  F2: 223.99 mg/dl (19.6) *  F3: 219.13 mg/dl (15.86) *  C: 126.33 mg/dl (13.86) *  1.42627 (0.15648) mmol/l  *P-value< 0.001 (compared to control, fresh malaria and repeated malaria)  **Raised** | - A significant increase in non-esterified fatty acids (NEFAs) were observed when the number of attacks was between 4-5 (p < 0.0001).  - a significant decrease in plasma phospholipids was observed p<0.0001 |
| *Krishna et al. 2009*  *(20329375)*  *English* | India | Cross-sectional observational study with 110 patients with malaria (both *P. falciparum* n=NR & *P. vivax* n=NR).  Day: “0” | 110 patients with malaria  *P. f.* n=NR  *P. v.* n=NR | “Controls were available in this study”, n= NR | Means and standard deviation | Mean mg/dl (SD), p-value  n=NR patients: 199.4 mg/dl (27.2) *  5.15648 (0.70339) mmol/l  n=NR controls: 130.0 mg/dl (28.6)*  3.3618 (0.7396) mmol/l  *P<0.05  Ref values: NR  **Raised** | Mean mg/dl (SD), p-value  n=NR patients: 36.16 mg/dl (5.04) *  0.9351 (0.13033) mmol/l  n=NR controls: 43.25 mg/dl (12.15) 1.11845 (0.3142) mmol/l* P< 0.05  **Lowered** | Mean mg/dl (SD), p-value  n=NR patients: 135.56 mg/dl (32.57) *3.50558 (0.84226) mmol/l  n=NR controls: 92.5 mg/dl (8.3)* 2.39205 (0.21464) mmol/l  P<0.05  *VLDL:*  ? patients: 79.6 mg/dl (3.6)*  ? controls: 76.9 mg/dl (8.8)*  *P<0.05  **Raised** | Mean mg/dl (SD), p-value  n=NR patients: 190.21 mg/dl (31.47)*  2.14747 (0.3553) mmol/l  n=NR controls: 148.25 mg/dl (16.59)* 1.67374 (0.1873) mmol/l  *P<0.05  **Raised** | - |
| *Njoku et al. 1995*  *(9163715)*  *English* | Nigeria | Cross-sectional observational study. Of 500 persons screened, 100 were positive for malaria.  Day: “0”and two weeks after treatment and recovery | 100 patients with severe *P. falciparum* infection | - | Means and standard deviation | Mean mg/100 ml, (SD)  100 *P. f.* Patients Mean: 157.5 (21.5)  4.07295 (0.55599) mmol/l during infection vs. mean: 174.1 (49.9) after infection. 4.50223 (1.29041) mmol/l  P-value: >0.01  **No sig. difference** | NR | NR | NR | -no sig. difference in free cholesterol levels during vs. after infection (mean 60.5 mg/100ml (SD 7.3) vs. 65.8 mg/100ml (SD19.5) P>0.01 |
| *Agbedana et al. 1990*  *(2256776)*  *English* | Nigeria | Cross-sectional observational study in childhood malaria.  Day: “0” | 41 in total  *Group I:* 15 *P. f.* patients | *Group II*  15 febrile patients without malaria, diagnosis NR  11 children without malaria and without symptoms (healthy controls) | Means and standard deviation | Means mg/100ml, (SD)  Group I*: 99 mg/100ml (27)  2.56014 (0.69822) mmol/l  Group II**:111 mg/100ml (27) 2.87046 (0.69822) mmol/l  Controls: 137 mg/100 ml (30) 3.54282 (0.7758) mmol/l  *Group I vs. controls: (t=3.3275, P<0.01  ** Group II vs. controls (t=2,2767, P<0.05)  **Lowered** | Means mg/100ml, (SD)  Group I*: 31 mg/100ml (11.6)  0.80166 (0.29998) mmol/l  Group II**:35 mg/100ml (10)  0.9051 (0.2586) mmol/l  Controls: 40 mg/100 ml (6.6)  1.0344 (0.17068) mmol/l  *Group I vs. controls (t=2.5029 P<0.02)  **P-value: NS  **Lowered** | NR | NR | -lowering of total cholesterol in groups I and II, but with a more pronounced change in group I patients with established *P. falciparum* malaria infection.  -HDL cholesterol showed a reduction in malaria patients more than in other febrile diseases. |
| *Seshadri et al. 1981*  *(7040221)*  *English* | India | Prospective observational study with patients with *P. vivax malaria*. Day “0”, D6 and after 6 weeks | 30 patients with *P. vivax* malaria. Treatment with: 16 cases with cholorquine/primaquine and 6 cases with “Ayurvedic antimalarial preperation”(traditonal Indian treatment) | 39 healthy controls | Means and standard error | Means mg/dl (SE), p-value  Day 0:  30 Patients: 109.0 (3.54)* 2.81874 (0.09154) mmol/l  39 Controls: 170.4 (4.3)* 4.40654 (0.1112) mmol/l  *P<0.001  Day 6:  22 patients: 126.1 (4.14) **3.26095 (0.10706) mmol/l  39 patients: NR  **P<0.02 (sig. diff between patients D6 and controls D0.  **Lowered** | NR | NR | NR | -increase of level of cholesterol and total lipids after treatment which was stat. Significant (D0: 109.0mg/dl (SE:3.54) vs D6: 126.1 mg/dl(4.14) P<0.02.  -there was a further sig. increase after 6 weeks (10 patients: 159.9 mg/dl (SE:6.49) vs. D0 & D6, P< 0.02. |
| *Maurois et al. 1979*  *(393192)*  *English* | France | Case-report of non-immune patient with *P. vivax* malaria  Day: “0”, D3, D5, D8, D10, D12, D14, D16, D18, D41 | 1 patient with *P. vivax* malaria and Morbus Buerger | - | Individual values | Total cholesterol lowers during acute attack from 1.6 g/l to 0.8 g/l  4.1376 mmol/l (2.0688)  **Lowered** | Decreased band of HDL on lipoproteinograms during malaria attack  **Lowered** | Marked bands of LDL, VLDL, IDL and chylomicrons during malaria attack  **Raised** | During the malaria attack the level of TG was three times recorded on the first day of infection.  **Raised** | - |
| *Chagnon et al. 1985*  *(PMID not available)*  *French* | France | Retrospective study in patients returning from the tropics.  Day: “0”  Total 67 cases, 46 hospitalized and of these 30 complete data.  ♂:♀ 26:4  15 Europeans  14 Africans  1 Pakistani | Total 30 malaria patients  20 *P. falciparum*  8 *P. vivax*  1 *P. ovale*  1 *P. malariae* | - | Frequency (%), means and ranges | Hypocholesterolemia (<1g/l) in 30/30 (100%) patients with acute malaria  15 Europeans  Mean: 3.09 mmol/l [1,89-4.03]  15 non-Europeans  Mean:2.94 mmol/l [1.41-4.4]  **Lowered** | Means:  Measurement 1: 0.40 mmol/l  Measurement 2: 0.31 mmol/l  Measurement 3: 0.28 mmol/l  Ref value: 0.77-1.5 mmol/l  **Lowered** | NR | NR | -Hypocholesterolemia (<1g/l) can be considered as suggestive of malaria.  - No sig. difference by plasmodium type observed |
| *Njoku et al 2001.*    *(11680094)*  *English* | Nigeria | Intervention study describing the effects of : “Azadirachta indica” (herbal medicine) on plasma lipid levels  Day: “0” and | Total 100 patients tested.  43 *P. f.*  Rx  Group 1: N=8 chloroquine, novalgin, panadol and vit B-complex  Group 2: N=25 *Azadirachta indica* | 22 controls; NR if healthy or symptoms | Means and standard deviation | Means mg/100ml, SD  Group 1: Mean 119.8 mg/100ml (20.6)  3.09803 mmol/l (0.53272) (8 patients tested)  Group 2: Mean 112.3 mg/100ml (11.7)  2.90408 mmol/l (0.30256)  (25 patients tested)  Weighted mean: 2.95109 (0.35453)  Controls: Mean 162.5 mg/100ml (19.2)  4.20225 mmol/l (0.49651)  P<0.01  **Lowered** | Means mg/100ml, SD  Group 1: Mean 75.3 mg/100ml (22.3)  1.94726 mmol/l (0.57668) (8 patients tested)  Group 2: Mean 82.1 mg/100ml (22.3)  2.12311 mmol/l (0.57668) (25 patients tested)  Weighted mean: 2.08047 (0.57668)  Controls: Mean 71.6 mg/100ml (23.1) 1.85158 mmol/l (0.59737)  P: NR  **Inconclusive** | Means mg/100ml, SD  Group 1: Mean 68.7 mg/100ml (31.2)  1.77658 mmol/l (0.80683) (8 patients tested)  Group 2: Mean 66.5 mg/100ml (30.6)  1.71969 mmol/l (0.79132) (25 patients tested)  Weighted mean:  1.73348 (0.79482)  Controls: Mean 114.6 mg/100ml (32.1)  2.96356 mmol/l (0.83011)  P<0.01  **Lowered** | Means mg/100ml, SD  Group 1: Mean 158.2 mg/100ml (24.5)  1.78608 mmol/l (0.27661) (8 patients tested)  Group 2: Mean 152.8 mg/100ml (26.9)  1.72511 mmol/l (0.3037) (25 patients tested)  Weighted mean: 1.73989 (0.29758)  Controls: Mean 118.2 mg/100ml (18.7)  1.33448 mmol/l (0.21112)  P<0.01  **Raised** |  |
| *Nicolas et al 1997.*    *(9091865)*  *French* | Gabon | Cross-sectional observational study in expatriates (French soldiers). Day:”0” | 154 *P. falciparum* patients  145 UCM patients  9 SM patients  Mean age: 23,7 y.  ♂: ♀ 153:1 | - | Frequency (%) and min-max. | Hypocholesterolemia in 18,5% (of 100 patients), min-max [1.93-5.8 mmol/l)  Cut off value: 2.58 mmol/l  Note: 72% used chloroquine -proguanil as profylaxis  **Lowered** | NR | NR | NR | - |
| *Das et al. 1996*  *(8669421)*  *English* | India | Cross-sectional study of Plasma alpha-tocopherol, retinol, and carotenoids in *children* with falciparum malaria. Cholesterol was measured as well. Day :0 | 100 total  *P. f.* (severe): 50  *P. f.* (mild): 50 | Healthy controls: N=50 | Means and standard deviation | Mean mmol/l, (SD), P-value  *50 P. f. (*severe*)* 1.89 (0.62)*  50 *P. f.* (mild)2.56 (0.62)*  (Mean weighed: 2.225 (0.62)  50 controls 3.47 (0.59) *  *P<0.001  **Lowered** | NR | NR | NR | - |
| *Selvam et al. 1992*  *(1459303)*  *English* | India | Prospective observational study in patients infected with *P. vivax* malaria  Day: “0” and after radical treatment (n=44)  Rx: chloroquine + primaquine | 152 *P. vivax* patients | 2 control groups:  -214 Healthy controls (no symptoms)  -44 “negative” controls with malaria like symptoms but no malaria | Frequency (%) Mean (SD) | Mean mg/dl, (SD)  90% of malaria patients had cholesterol level below 160mg/dl,  -98 malaria patients  Mean: 150.9 (40.1)* 3.90227 mmol/l (1.03699)  -28 negative controls  Mean: 173.6 (26.6)* 4.4893 mmol/l (0.68788)  -174 healthy controls  Mean:200.2 (32.2)  5.17717 mmol/l (0.83269)  *P<0.001  **Lowered** | NR | NR | NR | -free and ester cholesterols were sig. decreased (p<0.001) in malaria.  -serum cholesterol levels progressively decreased with increasing parasitemia, but the mean cholesterol levels were not sig. different from each other.  -Total cholesterol, ester and free cholesterol reached normal levels by D10 |
| *Mfonkeu et al. 2010*  *(20615271)*  *English* | Cameroon | Cross-sectional observational study to investigate biomarkers of nutritional status in childhood malaria  Day: “0” | 139 *P. f.* patients  94 *P. f.* (uncomplicated, “UCM” )  45 *P. f.* (cerebral) | 45 healthy controls | Means and standard error | Mean, g/l (SE), P-value  94 *P. f.*  (UCM ) 1.05 (0.05)  2.7153 mmol/l (0.1293)  45 *P. f.* (cerebral) 0.88 (0.06)*  2.27568 mmol/l (0.15516)  Total weighted mean: 2.57297 (139 patients) (0.13760)  45 controls 1.19 (0.08)  3.07734 mmol/l (0.20688)  P0.049  *P<0.05 sig. diff. from the value in the control group  **Lowered** | Mean, g/l (SE), P-value  94 *P. f.*  (UCM ) 0.42 (0.03) 1.08612 mmol/l (0.07758)  45 *P. f.* (cerebral) 0.34 (0.04) 0.87924 mmol/l (0.10344)  Total weighted mean: 1.01914 (0.08588)  45 controls 0.40 (0.04) 1.0344 mmol/l (0.10344)  P0.368  **No sig. difference** | Mean, g/l (SE), P-value  94 *P. f.*  (UCM ) 0.48 (0.30) 1.24128 mmol/l (0.7758)  45 *P. f.* (cerebral) 0.40 (0.24) 1.0344 mmol/l (0.62064)  Total weighted mean: 1.17430 (0.72596) mmol/l  45 controls 0.67 (0.36) 1.73262 mmol/l (0.93096)  P0.0753  **No sig. difference** | Mean, g/l (SE), P-value  94 *P. f.*  (UCM ) 0.49 (0.24) 0.55321 (0.27096) mmol/l  45 *P. f.* (cerebral) 0.57 (0.31) 0.64353 (0.34999) mmol/l  Total weighted mean: 0.58245mmol/l (0.29634)  45 controls 0.52 (0.24) (0.58708 mmol/l (0.27096 )  P0.3624  **No sig. difference** | - |
| *Ayoola et al 2012*  *(22429464)*  *English* | Nigeria | Cross-sectional observation study in pregnant woman with malaria.  Day: “0” in trimester 2 and 3 | Total *P. f.* patients (pregnant) in trimester 2=17 and in trimester 3=55. | Healthy controls in trimester 2= 93  Healthy controls in trimester 3=231 | Median, IQ Range | Medan, [range]  *Trimester 2:*  17 *P. f.* Mean: 3.73 mmol/l [2.80-4.28]  93 controls Mean: 4.73 mmol/l [4.26-5.71]  P-value:<0.001  *Trimester 3:*  55 *P. f.* Mean: 4.43 mmol/l [3.67-5.41]  231 controls Mean:5.17 mmol/l [4.50-5.81]  P-value: <0.001  Ref value: 3-5 mmol/l  **Lowered** | Medan, [range]  *Trimester 2:*  17 *P. f.* Mean: 0.86 mmol/l [0.70-1.38]  93 controls Mean: 1.58 mmol/l [1.37-2.02]  P-value: <0.001  *Trimester 3:*  55 *P. f.* Mean: 1.27 mmol/l [0.97-1.63]  231 controls Mean:1.64 mmol/l [1.39-1.84]  P-value: <0.001  Ref value: 1.2-2.2 mmol/l  **Lowered** | Medan, [range]  *Trimester 2:*  17 *P. f.* Mean: 2.10 mmol/l [1.51-2.53]  93 controls Mean: 2.66 mmol/l [2.03-3.28]  P-value:0.010  *Trimester 3:*  55 *P. f.* Mean: 2.44 mmol/l [1.70-3.08]  231 controls Mean:2.77 mmol/l [2.31-3.43]  P-value: 0.006  Ref value: 2-3 mmol/l  **Lowered** | Medan, [range]  *Trimester 2:*  17 *P. f.* Mean: 1.35 mmol/l [1.19-1.62]  93 controls Mean: 1.29 mmol/l [1.02-1.51]  P-value:0.112  *Trimester 3:*  55 *P. f.* Mean: 1.51 mmol/l [1.28-1.99]  231 controls Mean:1.47 mmol/l [1.24-1.77]  P-value: 0.125  Ref value: 0.6-1.68 mmol/l  **No sig. difference** | - |
| *Ramírez-Olivencia et al 2012*  *(22036173)*  *Spanish* | Spain | Cross-sectional, cohort and retrospective study with imported malaria  Day: “0” | 398 malaria patients (50% natives of endemic areas, 50% travellers) | - | Frequency (%) | Frequency (%)  16,2% of 121 malaria patients vs. 5% of 277 non-malarious patients with hypocholesterolemia P-value: NR  Ref-value: NR  **Inconclusive** | NR | NR | NR | - |
| *Hansmann et al. 1997*  *(9410545)*  *French* | France | Retrospective study in 210 hospitalized patients with malaria returning from the tropics.  Day: “0”  49% French pt  44.8% Africans  6.2% Other | Total: 210 patients  160 *P. f.*  18 *P. v.*  15 *P. o.*  5 *P. m.*  4 *P. f. + P. m.*  2 *P. f. + P. o.*  2 *P. f. + P. v.*  4 NR | - | Frequency (%), means and standard deviation | 170 patients (81%) with a hypocholesterolaemia.  Mean (for all patients) : 3.04 mmol/l (SD : 0.93)  Africans (n= 94)  Mean: 2.86 mmol/l  Europeans (n=116)  Mean: 3.19 mmol/l  P-value: <0.01  **Lowered** | NR | NR | 130 patients of 210 patients (62%) with hypertriglyceridemia  Mean : 2.3 mmol/l (SD : 1.46).  Severe group (WHO definition) (n=15)  Mean : 3.054 mmol/l  Non severe group (n=195)  Mean: 2.29 mmol/l  P-value: <0.01  **Raised** | -Hypertriglyceridemia seems to be associated with severe malaria  -Hypocholesterolemia more striking in African subjects, but also observed in Europeans (nutritional factors and ethnicity alone cannot explain this difference) |
| *Alcenar et al. 2001*  *(PMID not available)*  *Portuguese* | Brazil | Cross-sectional study to assess nutritional and dietary status of malaria patients in the Amazones  Day: “0” | 268 malaria patients *P. falciparum* and/or *P. vivax*  ♂:♀ 198:70 | - | Means (SD) | Mean (mg/dl), SD  Hypocholesterolemia 100% (121/121) in every age group.  121 *P. f.* patients  Mean: 29.7 mg/dl (18.8) 0.76804 mmol/l (0.48617)  Ref value: NR  P<0.05  **Lowered** | NR | NR | Mean (mg/dl), SD  121 *P. f.* patients  Mean: 120.8 mg/dl (81.3)  Ref value: NR  P>0.05  1.36383 (0.91788) mmol/l  **No sig. difference** | - |
| *Eteng et al. 2010*  *(PMID not available) WOS: 000278381200026)*  *English* | Nigeria | Cross-sectional observational study with pregnant malaria patients.  Day:”0” | 17 *P. falciparum* pregnant patients | 20 healthy (pregnant) controls | Means (SD) | Means mmol/l, SD  17 pregnant *P.f.* Mean: 4.28 mmol/l (1.16)  20 healthy pregnant controls  Mean:2.95 mmoll (0.71)  P-value:<0.01  **Raised** | NR | NR | Means mmol/l, SD  17 pregnant *P.f.* Mean: 1.56 mmol/l (0.14)  20 healthy pregnant controls  Mean:1.17 mmol/l (0.28)  P-value: <0.01  **Raised** | - |
| *Ogbodo et al. 2008*  *(PMID not available, accession number: 2008420252)*  *English* | Nigeria | Cross-sectional observational study in childhood malaria.  Day: “0” | 20 *P. f.* patients | 15 age-matched, uninfected children | Means (SD) | Means (mg/100ml) (SD)  20 *P. f.* patients Mean:87.8 mg/ 100ml (16.7) 2.27051 mmol/l (0.43186)  15 healthy controls Mean:111.2 mg/100ml (22.5) 2.87563 mmol/l (0.58185)  P-value: 0.0027  **Lowered** | Means (mg/100ml) (SD)  20 *P. f.* patients Mean: 36.5 mg/ 100ml (10.4)  0.94389 mmol/l (0.26894)  15 healthy controls Mean:24.2 mg/100ml (7.9)  0.62581 mmol/l (0.20429)  P-value: 0.0013  **Lowered** | Means (mg/100ml) (SD)  20 *P. f.* patients Mean:53.2 mg/ 100ml (14.4) 1.37575 mmol/l (0.37238)  15 healthy controls Mean:46.0 mg/100ml (16.3) 1.18956 mmol/l (0.42152)  P-value: 0.2493  **Lowered** | Means (mg/100ml) (SD)  20 *P. f.* patients Mean: 108 mg/ 100ml (32.9)  1.21932 mmol/l (0.37144)  15 healthy controls Mean:88.9 mg/100ml (22.8) 1.00368 mmol/l (0.25741)  P-value: 0.0694  **Raised** | -no sig. correlation (p>0.05) between parasite density and the levels of lipids and lipoproteins, however, relative decreases were observed as the parasitemia increased. There was a sig. diff (p<0.05) if highest was compared with the lowest density |
| *Ozkaya et al. 2006*  *(17205701)*  *Turkish* | Turkey | Case report of expatriate returning from Mozambique with *P. falciparum* malaria  Day: NR | 1 *P. f.* patient | - | Individual values | Hypocholesterolemia of 2 mg/dl  0.05172 mmol/l  Ref value: NR  **Lowered** | NR | NR | NR | - |
| *Al-Omar et al.*  *2010*  *(WOS:000280757600012*  *English* | Saudi Arabia | Prospective observational study in *Plasmodium falciparum* infection.  Day: “0” | 200 *P. f.* patients | 200 healthy blood donors matched for age and sex from non-endemic areas | Means (SD), (SE) | Means (mmol/l) (SD)  200 *P .f.* patients.  Mean: 1.93 mmol/l (SD:1.59) (SE:0.11)  Range 0.2-6.3  200 Healthy controls  Mean: 3.60 mmol/l  (SD:0.99) (SE: 0.069) Range 1.4-5.2  P<0.001 (malaria vs. controls)  **Lowered** | NR | NR | NR | -a clear and significant inverse correlation between parasite count and cholesterol level among the patients (Pearson correlation: 0.452) |
| *Blair et al.*  *2002*  *(11858128)*  *Spanish* | Columbia | Prospective cross-sectional study to study links between nutrition and immunity in children with or without malaria  Day: “0” | 51 *P. f.* patients | 49 healthy children without symptoms and negative malaria thick blood film | Mean (SD) | NR | NR | NR | NR | Apolipoprotein A1  26 boys with *P. f.*  malaria: Mean: 68 mg/dL (SD 25)  23 healthy boys  Mean:99 mg/dL (SD 15)  Ref value: 94-178 mg/dL boys  P-value: 0.000004  25 girls with *P. f.* malaria, Mean: 71 mg/dL (SD 24)  26 healthy girls  Mean: 102 mg/dL, (SD 14)  P-value: 0,8 x 10^–8^  Ref. Value: 101-198 mg/dL girls  **Lowered** |
| *Vinoth et al.*  *2011*  *(21934231)*  *English* | India | Case report of hemophagocytic syndrome associated with *Plasmodium falciparum* infection  Day: “0” | 1 *P. f.*  patients | n/a | NR | NR | NR | NR | Patient presented with a “hypertriglyceridemia”. | - |
| *D’Souza et al.*  *2012*  *English* | India | FULL TEXT REQUESTED,  NOT RETRIEVABLE | ? | ? | ? | ? | ? | ? | ? | Titled: “Influence of malaria on high density lipoprotein cholesterol and total cholesterol/HDL cholesterol ratio in two cases of diabetes mellitus” |
| **Source (first author, year of publication)**  **(PubMed ID)**  ***Language*** | **Country (study site)** | **Study-design + Day of sample collection** | **No. of participants with malaria** | **No. of controls** | **Measure of outcome** | **Total cholesterol (TC)** | **High-density lipoprotein cholesterol (HDL)** | **Low-density lipoprotein cholesterol (LDL)** | **Triglycerides (TG)** | **Other findings** |

**Additional file 7: Table S1. Studies included in the systematic review and meta-analysis.** “Judgment” (**bold**/colour)= conclusion for [parameter] of malaria patient vs. reference value, malaria group vs. control group or before/during treatment vs. after treatment. First author; year of publication; Journal; Country = study site; Study-design, day of sample collection: “0”is defined as “blood collection at admission, before treatment”; no. of patients included with malaria, and if available, described for different species; no. of controls, and if available, description of this group; total-cholesterol, different reference values are used, if available, reported. Units are different and specified. NR= not reported. NS= not significant. “Sig.”= significant “Diff.” = difference. SD=standard deviation SE=standard error OR= Odds Ratio. UCM= Uncomplicated malaria, SM= severe malaria

Online conversion tool: <http://www.onlineconversion.com/cholesterol.htm> (last accessed 10 September 2013)
