# Supplementary material for: Serum lipids and lipoproteins in malaria - a systematic review and meta-analysis
Source: Malar J. 2013 Dec 7;12:442. doi: 10.1186/1475-2875-12-442 (PMC4029227; doi:10.1186/1475-2875-12-442)
Supplement: Additional file 8 — Forest plots without the study of Faucher et al. The data provided describes the results of the meta-analysis for the different lipid parameters investigated without one study with a control group consisting of (very) low parasitaemia malaria patients. [file 1475-2875-12-442-S8.doc]

**Additional File 7:** Forest plots without the study of Faucher et al. 2002 (1)

Forest plot. Outcome: cholesterol. Malaria patients vs. healthy controls. Random effect model.

Forest plot. Outcome: HDL. Malaria patients vs. healthy controls. Random effect model.

Forest plot. Outcome: LDL. Malaria patients vs. healthy controls. Random effect model.

Forest plot. Outcome: triglycerides. Malaria patients vs. healthy controls. Random effect model.

**References**

1. Faucher JF, Ngou-Milama E, Missinou MA, Ngomo R, Kombila M, Kremsner PG: **The impact of malaria on common lipid parameters.** *Parasitol Res* 2002, **88:** 1040-1043.
